# Comparative analyses of CTCF and BORIS occupancies uncover two distinct classes of CTCF binding genomic regions

**DOI:** 10.1186/s13059-015-0736-8

**Published:** 2015-08-14

**Authors:** Elena M. Pugacheva, Samuel Rivero-Hinojosa, Celso A. Espinoza, Claudia Fabiola Méndez-Catalá, Sungyun Kang, Teruhiko Suzuki, Natsuki Kosaka-Suzuki, Susan Robinson, Vijayaraj Nagarajan, Zhen Ye, Abdelhalim Boukaba, John E. J. Rasko, Alexander V. Strunnikov, Dmitri Loukinov, Bing Ren, Victor V. Lobanenkov

**Affiliations:** Molecular Pathology Section, Laboratory of Immunogenetics, National Institute of Allergy and Infectious Diseases, National Institutes of Health, Rockville, MD 20852 USA; Ludwig Institute for Cancer Research, 9500 Gilman Drive, La Jolla, CA 92093 USA; Department of Cellular and Molecular Medicine, Institute of Genomic Medicine, Moores Cancer Center, San Diego School of Medicine, University of California, San Diego, La Jolla, CA 92093 USA; Bioinformatics and Computational Biosciences Branch, Office of Cyber Infrastructure and Computational Biology, National Institute of Allergy and Infectious Diseases, National Institutes of Health, Bethesda, MD 20892 USA; Stem Cell Project, Tokyo Metropolitan Institute of Medical Science, Kamikitazawa, Setagaya-ku, Tokyo Japan; Guangzhou Institutes of Biomedicine and Health, Molecular Epigenetics Laboratory, 190 Kai Yuan Avenue, Science Park, Guangzhou, 510530 China; Gene and Stem Cell Therapy Program, Centenary Institute, Camperdown, NSW 2050 Australia; Sydney Medical School, University of Sydney, Sydney, NSW 2006 Australia; Cell and Molecular Therapies, Royal Prince Alfred Hospital, Camperdown, NSW 2050 Australia

## Abstract

**Background:**

CTCF and BORIS (CTCFL), two paralogous mammalian proteins sharing nearly identical DNA binding domains, are thought to function in a mutually exclusive manner in DNA binding and transcriptional regulation.

**Results:**

Here we show that these two proteins co-occupy a specific subset of regulatory elements consisting of clustered CTCF binding motifs (termed 2xCTSes). BORIS occupancy at 2xCTSes is largely invariant in BORIS-positive cancer cells, with the genomic pattern recapitulating the germline-specific BORIS binding to chromatin. In contrast to the single-motif CTCF target sites (1xCTSes), the 2xCTS elements are preferentially found at active promoters and enhancers, both in cancer and germ cells. 2xCTSes are also enriched in genomic regions that escape histone to protamine replacement in human and mouse sperm. Depletion of the BORIS gene leads to altered transcription of a large number of genes and the differentiation of K562 cells, while the ectopic expression of this CTCF paralog leads to specific changes in transcription in MCF7 cells.

**Conclusions:**

We discover two functionally and structurally different classes of CTCF binding regions, 2xCTSes and 1xCTSes, revealed by their predisposition to bind BORIS. We propose that 2xCTSes play key roles in the transcriptional program of cancer and germ cells.

**Electronic supplementary material:**

The online version of this article (doi:10.1186/s13059-015-0736-8) contains supplementary material, which is available to authorized users.

## Background

CTCF, a highly conserved DNA binding protein, serves as a global organizer of chromatin architecture [1]. It is involved in the regulation of transcriptional activation and repression, gene imprinting, control of cell proliferation and apoptosis, chromatin domain insulation, X-chromosome inactivation, prevention of oligonucleotide repeat expansion, and other chromatin resident processes [2–11]. The multifunctionality of CTCF is based on its ability to bind a wide range of diverse DNA sequences as well as to interact with cofactor proteins through the combinatorial use of 11 C2H2 zinc fingers (ZFs) [12–15]. With the advance of next-generation sequencing techniques, CTCF binding sites have been identified across fly, mouse, and human genomes [14, 16, 17]. The genome-wide studies helped defined the DNA binding specificity of CTCF, known as CTCF target sites (CTSes) [1, 13, 18]. CTSes tend to be conserved in evolution and occupancy is largely invariant across different cell types. Reflecting the multitude of CTCF functions, CTSes were found to be associated with the genomic regions engaged in long-range chromatin interactions, including enhancers [19], promoters [14], insulators [20] and boundary elements [8]. The capacity of CTCF–DNA complexes to form loops via protein dimerization as originally described for the H19-IFG2 imprinted locus [21] has been confirmed genome-wide by three-dimensional approaches, solidifying the key role of CTCF in the organization of chromatin architecture [7, 22]. For example, CTCF-mediated chromatin loops were shown to connect enhancers with promoters [19], to insulate promoters from enhancers [23], to mediate imprinting of mammalian genes [24], to control V(D)J recombination [25], and to organize the major histocompatibility complex (MHC) class II genes [26]. It remains obscure, however, how the DNA sequences of given CTSes are related to the specific CTCF functions at these sites.

CTCF gene duplication during early evolution of amniotes gave rise to Brother Of the Regulator of Imprinting Sites (BORIS) [27, 28]. CTCF and BORIS encode proteins that share an almost identical DNA binding domain recognizing the same DNA sequences in vivo and in vitro [29–32]. It has long been thought that CTCF and BORIS possess distinct functions and act in a mutually exclusive manner. Indeed, while CTCF is ubiquitously expressed, BORIS expression is strictly restricted to germ cells in normal development [27]. However, BORIS is aberrantly expressed in a wide range of cancers, and its function in that context has not been characterized [31, 33–36]. To date, established BORIS functions are limited to the transcriptional activation or repression of some germline and cancer-related genes [29, 30, 32]. Due to the completely distinct amino and carboxyl termini of CTCF and BORIS proteins, differences in biological functions between the two factors were expected. This was supported by the contrasting phenotypes of their germline knockouts as well as by the inability of BORIS to complement CTCF mutations [29, 30, 37]. The homozygous deletion of CTCF in mice showed early embryonic lethality at the peri-implantation stage [37]. In contrast, BORIS knockout mice showed subfertility and multiple defects in spermatogenesis, including a reduction in testis size and delayed production of gametes [29, 30].

The fact that CTCF and BORIS share a virtually identical DNA binding domain and are co-expressed in at least two environments, in germ and cancer cells [13], raises the question of whether they bind competitively or cooperatively at a given DNA sequence [13, 27, 38]. It has been proposed that CTCF and BORIS compete for DNA binding with the complete replacement of one protein by the other at target sequences [27, 30]. This model predicts disruption of CTCF function in cancer cells or in germ cells. Given the important function of CTCF as a genome-organizer, however, the above model would also predict global disruption of genome organization and consequently large-scale changes in gene expression patterns. To address this problem, we have developed and utilized a set of monoclonal and polyclonal antibodies to map CTCF and BORIS binding sites in both human and mouse genomes. We report here, for the first time, CTCF and BORIS occupancy of chromatin in germ cells and in several cancer cell types. We found that BORIS, together with CTCF, occupies as much as one-third of CTSes and “sidesteps” the remaining two-thirds of CTSes regardless of the origin of cancer cells. We demonstrate that the pattern of BORIS occupancy in cancer cells expressing this protein largely recapitulates its binding in germ cells, suggesting that the ability of a CTCF binding region to be occupied by BORIS in vivo is encoded in the DNA sequence and the site’s architecture. We further show that CTCF and BORIS bound regions (CTCF&BORIS) typically contain at least two proximal CTSes (2xCTS) in germ and cancer cells. 2xCTS elements are preferentially found at active promoters and enhancers, and are associated with retained histones in human and mouse sperm, in stark contrast to genomic regions harboring a single CTS (1xCTS). Our results also establish the functional significance of 2xCTSes in cell-specific gene expression.

## Results

### BORIS selectively binds to a subset of CTCF binding regions in cancer cells regardless of tissue origin

**Fig. 1:**
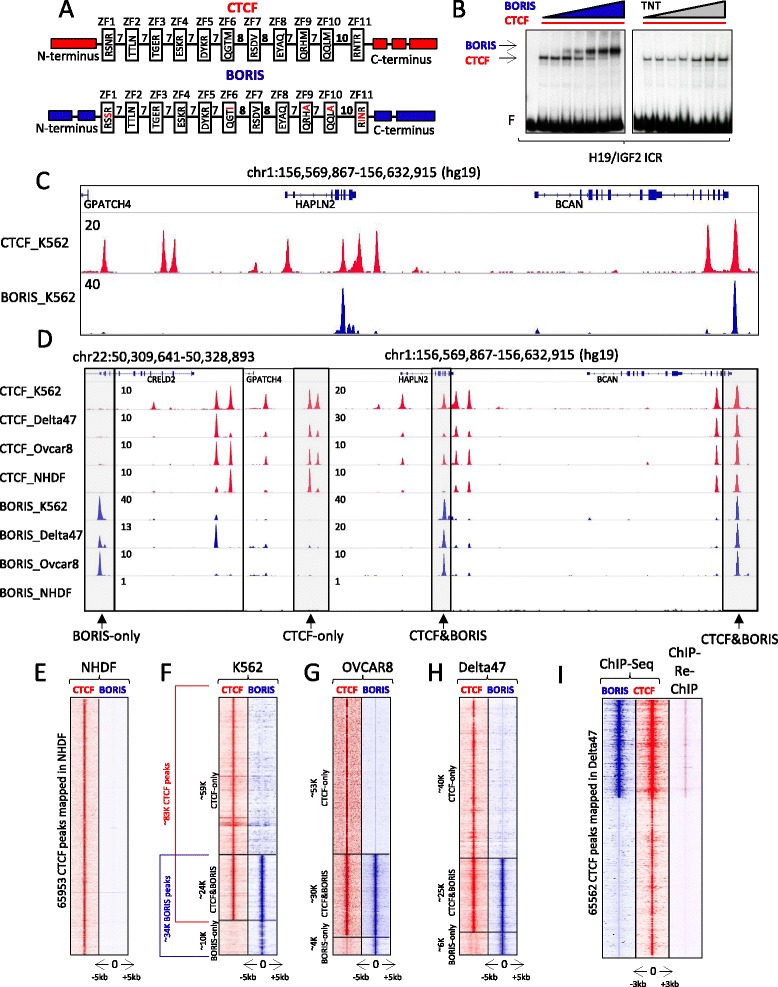
Genomic profiling of CTCF and BORIS occupancy in human cancer cells. **a** Schematic representation of CTCF and BORIS zinc fingers (*ZF*) shows the four essential amino acids involved in DNA recognition for each ZF. The amino acids of BORIS that differ from CTCF are highlighted in *red*. The numbers between ZFs show linker length. **b** Electrophoretic mobility shift assay (EMSA) with increasing amount of full-length BORIS protein (*left panel*) and unprogrammed TNT (*right panel*) on the background of a constant level of full-length CTCF protein. The fourth CTCF binding site out of seven mapped in the H19-IGF2 imprinting control region (*ICR*) was used as the labeled probe. **c**, **d** Gene tracks show the distribution of CTCF (*red*) and BORIS (*blue*) bound regions in the K562 cell line (**c**) and in cell lines K562, Delta47, OVCAR8, and normal human dermal fibroblasts (*NHDFs*) (**d**). The molecules against which antibodies were directed and cell lines used in ChIP-seq are shown on the *left*. The number of tags per one million of mapped reads is indicated. The gray frames and arrows show CTCF-only, CTCF&BORIS and BORIS-only bound regions. **e**–**h** Heatmaps depict CTCF (*red*) and BORIS (*blue*) occupancy in NHDFs (**e**), K562 (**f**), OVCAR8 (**g**) and Delta47 (**h**) cells. **e** Heatmap shows the absence of BORIS occupancy at CTCF bound regions mapped in BORIS-negative NHDFs. **f** The genome-wide overlapping of CTCF- and BORIS-bound regions mapped in K562 cells is shown on the *left side* of the heatmap. The overlapping of CTCF and BORIS occupancy was done independently for each cell type. The tag density of CTCF and BORIS ChIP-seq data was collected within a 10-kb window around the summit of CTCF (CTCF&BORIS and CTCF-only) and BORIS peaks (BORIS-only). The collected data were subjected to k-means clustering using linear normalization based on similar tag density profiles. The molecules against which antibodies were directed in ChIP-seq are listed on top of the heat map. **i** Heatmap shows the enrichment of ChIP-Re-ChIP tag density at CTCF&BORIS-bound regions. ChIP-Re-ChIP occupancy (*purple*) is presented in comparison with CTCF (*red*) and BORIS (*blue*) ChIP-seq data for Delta47 cells. The tag density was subjected to k-means ranked clustering with two clusters expected

CTCF and BORIS share a conserved 11 ZF DNA binding domain (Fig. 1a) and, as a result, are identical in DNA binding specificity in vitro [27, 32, 39]. In vitro assays on single CTCF binding sites indicate that CTCF and BORIS compete for DNA binding with the complete replacement of one protein by the other at target sequences (Fig. 1b). It is unclear, though, whether the two proteins would compete for binding to the same genomic regions in vivo. To better understand the functional interplay between CTCF and BORIS in human cells, we performed ChIP-seq in three cancer cell lines — K562 (chronic myelogenous leukemia line), Delta47 (multiple myeloma line), and OVCAR8 (ovarian cancer line) — all of which express comparable levels of CTCF and BORIS proteins in the nucleus (Fig. S1a–c in Additional file 1). For this purpose, we developed a large set of anti-CTCF and anti-BORIS mouse monoclonal antibodies, thoroughly validated by immunoblotting, electrophoretic mobility shift assay (EMSA), and ChIP-seq for both BORIS-positive cancer cells and BORIS-negative normal human dermal fibroblasts (NHDFs) (Fig. 1c–h; Fig. S1b-d in Additional file 1). The patterns of BORIS and CTCF occupancy were very similar among the three cancer cell lines despite their distinct tissue origins (Fig. 1d–h; Fig. S1e, f in Additional file 1). Contrary to the expectation of overlapping CTCF and BORIS occupancy at all CTCF binding regions, we observed that only a subset (~29–38 %) of CTCF binding regions were also occupied by BORIS (designated here as CTCF&BORIS bound regions). Additionally, in each tumor cell line we found a small number of regions occupied by BORIS alone (BORIS-only bound regions), but the same regions were occupied by CTCF in other cell lines (Fig. S1f–h in Additional file 1). Importantly, the majority of the CTSes were not bound by BORIS in vivo (CTCF-only bound regions).

The model of simple competition between CTCF and BORIS in vitro (Fig. 1b) would predict lower CTCF and BORIS occupancy at CTCF&BORIS bound regions compared with CTCF-only and BORIS-only bound regions. Instead, the ChIP-seq peak intensity (tag density) for the coinciding CTCF and BORIS peaks was significantly higher compared with CTCF-only and BORIS-only peaks, respectively (Fig. S2a in Additional file 2). ChIP-Re-ChIP assay in K562 and Delta47 cells further confirmed that these CTCF&BORIS co-occupied regions were simultaneously bound by both proteins (Fig. 1i; Fig. S2b in Additional file 2), while CTCF-only and BORIS-only bound regions lacked the co-occupancy (Fig. S2b in Additional file 2). Taken together, the above evidence suggests that CTCF bound regions detected by the ChIP-seq approach are not homogeneous and can be subdivided into at least two groups based on their potential to be also occupied by BORIS.

### The genomic regions co-occupied by CTCF and BORIS proteins contain clustered CTCF binding sites

**Fig. 2:**
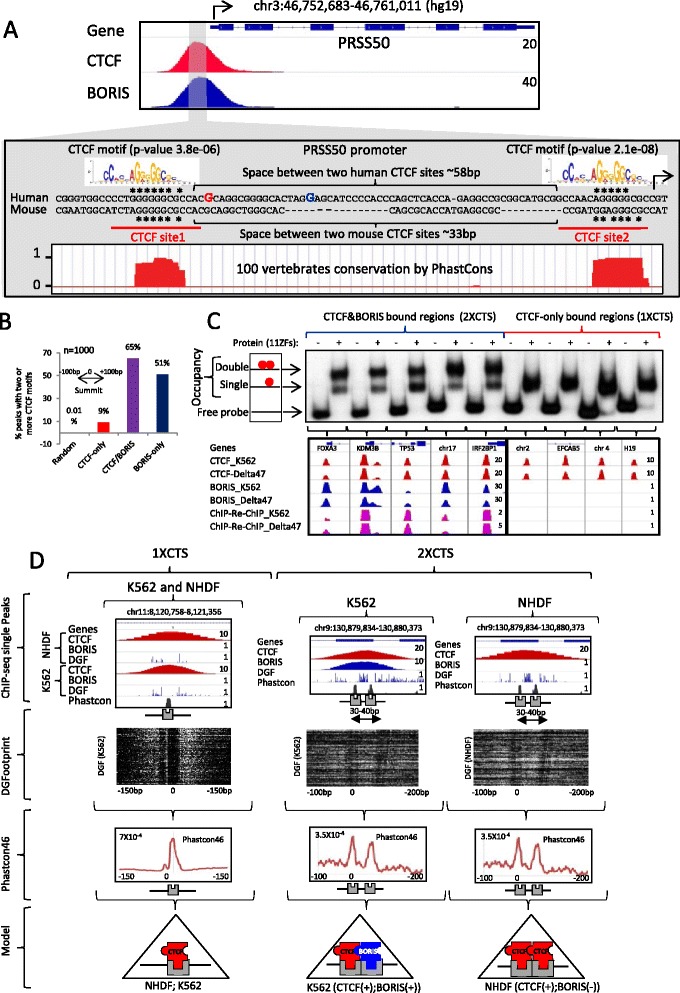
CTCF&BORIS bound regions enclose at least two closely located CTCF binding sites. **a** *Upper panel*: gene track represents CTCF (*red*) and BORIS (*blue*) binding at the *TSP50* (*PRSS50*) promoter in K562 cells. *Lower panel*: the alignment of human and mouse sequences under the summit of CTCF (*red nucleotide*) and BORIS (*blue nucleotide*) peaks mapped by ChIP-seq at the *TSP50* promoter. Two CTCF core motifs (shown on the *top* of the alignment) coincide with two CTCF binding sites (*site1* and *site2*) previously identified by EMSA and methylation interference assay (MIA) [32] in both mouse and human *TSP50* promoters (labeled and underlined at the *bottom* of the alignment). *Asterisks* denote the contact guanines mapped by MIA. The space between the two CTCF binding motifs in human and mouse *TSP50* promoters varies from 33 bp to 58 bp (shown by *brackets*). The phastCons conservation track shows the high conservation of two CTCF sites in the *TSP50* promoter among 100 vertebrates. The *black arrow* shows the beginning of *TSP50* transcription. **b** The percentage of CTCF-only, CTCF&BORIS and BORIS-only peaks with two or more CTCF motifs. The top 1000 CTCF-only, CTCF&BORIS and BORIS-only binding regions (invariant in three cancer cell lines) were selected for analysis. The presence of a CTCF motif was calculated by FIMO (MEME suite) in the sequence extended 100 bp upstream and downstream of the summit of either CTCF (CTCF-only and CTCF&BORIS) or BORIS (BORIS-only) peaks. Each CTCF motif occurrence has a *p* value < 0.0001. **c** *Upper panel*: EMSA with five CTCF&BORIS (*blue bracket*) and four CTCF-only (*red bracket*) binding regions. The ~200-bp ^32^P-labeled probes were incubated with either in vitro translated luciferase (−) or with the 11 ZF domain of CTCF (+). The slower (shown by *arrow with two red dots*) and faster (*arrow with one red dot*) migrating shifted bands correspond to CTCF binding to two CTCF sites at once or to one CTCF site, respectively (double and single occupancy). *Lower panel*: genome browser view of CTCF and BORIS occupancy in K562 and Delta47 cells at nine DNA sequences used in the EMSA. The *brackets* show the connection between upper and lower panels. ChIP-seq data are shown in combination with ChIP-Re-ChIP-seq data for K562 and Delta47 cells. ChIP-seq tracks are labeled with the molecule against which antibodies were directed and the cell line used. **d** *First row*: individual examples of 1xCTS and 2xCTS, differentially occupied by CTCF and BORIS in BORIS-positive (K562) and BORIS-negative (NHDF) cells. CTCF and BORIS ChIP-seq data are combined with digital genomic footprinting (*DGF*, ENCODE data) and phastCons46 conservation scores. The core 20-bp CTCF motif is marked by a *grey box. Second row*: heatmaps show DNaseI cleavage density at thousands of 1xCTSes (single CTCF motif on plus strand) and at hundreds of 2xCTSes (two CTCF motifs separated by a 30–40-bp linker, both on the minus strand). The tag density data were collected within a 300-bp window around the first (*left*) 20-bp CTCF core motifs (0) under a single CTCF ChIP-seq peak. *Third row*: average phastCons46 conservation score at 1xCTSes and 2xCTSes, the same genomic regions as in the second row. Fourth row: model of CTCF and BORIS differential occupancy in NHDF and K562 cells. 1xCTSes are occupied by CTCF monomer in both BORIS-positive and BORIS-negative cells, while 2xCTSes are co-occupied by CTCF and BORIS in BORIS-positive cells or by CTCF homodimer in BORIS-negative cells

In order to determine whether DNA sequences per se were responsible for the selective BORIS co-localization with CTCF*,* we performed EMSA with three classes of sequences: CTCF&BORIS, CTCF-only and BORIS-only bound regions. We observed that full-length CTCF and BORIS proteins bind the target sequences with similar affinity in vitro (Fig. S3 in Additional file 3). Unexpectedly, EMSA with probes corresponding to CTCF&BORIS and BORIS-only bound regions demonstrated a double shift with the in vitro translated 11 ZF domain (Fig. S3a, c in Additional file 3), indicating the presence of two adjacent CTCF binding sites in keeping with the demonstration that the same arrangement exists in the promoter of the *TSP50* (*PRSS50*) gene [32]. Furthermore, the testis-specific *TSP50* promoter is occupied by both CTCF and BORIS proteins in all three human cancer cell lines, thereby representing a bona fide CTCF&BORIS bound region with two conserved individual CTCF sites located 58 and 33 base pairs (bp) apart in the human and mouse promoters, respectively (Fig. 2a). The contact guanine residues, mapped for CTCF and BORIS binding at the *TSP50* promoter [32], coincided with two 20-bp CTCF binding motifs. Moreover, the CTCF motif was found to be represented at both CTCF and BORIS ChIP-seq peaks (Fig. 2a; Fig. S4a in Additional file 4). Therefore, we examined other CTCF&BORIS bound regions for the hidden presence of two CTCF binding sites utilizing the predictive power of the double CTCF motif feature detected in the *TSP50* promoter (Fig. 2a). Strikingly, 65 % of CTCF&BORIS and 51 % of BORIS-only bound regions consist of at least two closely spaced CTCF motifs, while CTCF-only bound regions generally (91 %) have only one CTCF motif under the peak (Fig. 2b; Fig. S4b–f in Additional file 4). To further validate this observation, we screened several randomly picked genomic loci representing either CTCF&BORIS or CTCF-only bound regions by EMSA (Fig. 2c; Fig. S5a in Additional file 5). All 12 CTCF&BORIS bound regions produced the double shifts with the 11 ZF domain, indicating the presence of two CTCF binding sites (Fig. 2c; Fig. S5a in Additional file 5), while all 11 CTCF-only bound regions produced a single binding shift pointing to a single CTCF binding site inside the sequences (Fig. 2c; Fig. S5a in Additional file 5). One could hypothesize that in the cases where a second motif for a dual CTCF&BORIS bound region was not identified, it is most likely still present but deviates from the CTCF motif sequence. Indeed, five such CTCF&BORIS bound regions produced double shifts with the 11 ZF domain, confirming the presence of two CTCF binding sites (Fig. S5a in Additional file 5). Therefore, we classified CTCF binding regions in the genome into clustered CTSes (2xCTSes) and single/individual CTSes (1xCTSes). We further validated the 2xCTS model by separating the two sub-sites of CTCF&BORIS bound regions and demonstrating their independent binding to the 11 ZF domain (Fig. S5b in Additional file 5).

The above data are consistent with the model of differential occupancy of CTCF bound regions by BORIS (Fig. 2d). Namely, 1xCTSes and 2xCTSes could not be discriminated at the resolution of ChIP-seq experiments and were detected, therefore, as single CTCF binding sites in both BORIS-positive (K562) and BORIS-negative (NHDF) cells (Fig. 2d). In BORIS-positive cells, the 2xCTSes were preferentially occupied by both CTCF and BORIS proteins (CTCF&BORIS), while 1xCTSes were preferentially occupied only by CTCF (CTCF-only) (Fig. 2d), thus revealing the two classes of CTCF binding regions. To further test our model, we analyzed DNaseI digital genomic footprinting and phastCons46 conservation score at 1xCTS and 2xCTS binding regions (Fig. 2d; Fig. S6 in Additional file 6). Upon plotting DNaseI cleavage density and the conservation score across the two classes of CTCF binding regions, we observed either single or double footprints, respectively, with the corresponding single or double conserved peaks, respectively (Fig. 2d; Additional file 6). As 2xCTSes produced double footprints in BORIS-negative (NHDF) cells as well as in BORIS-positive cells, we could assume that the 2xCTSes were occupied by CTCF homodimers in the absence of BORIS (Fig. 2d; Additional file 6).

### Clustered CTCF binding sites facilitate CTCF and BORIS interactions on DNA

**Fig. 3:**
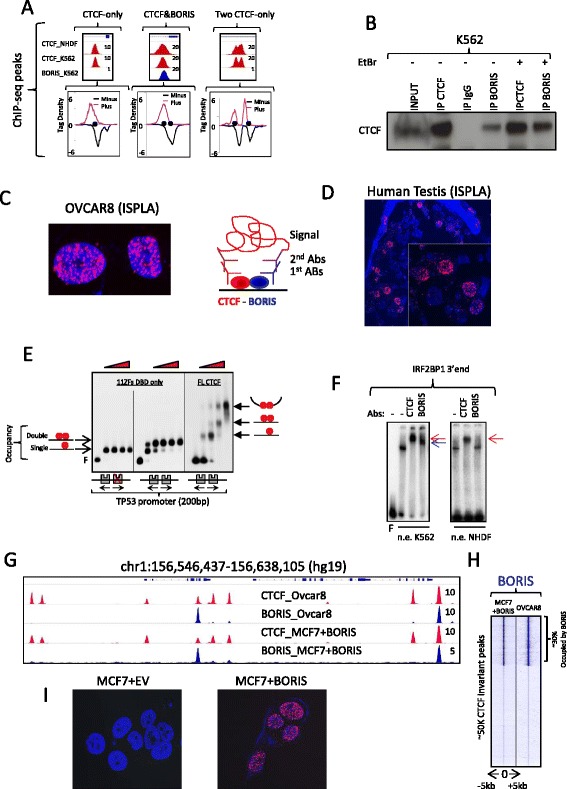
CTCF and BORIS interact at CTCF&BORIS bound regions. **a** ChIP-seq does not resolve the two closely spaced CTCF binding sites at CTCF&BORIS bound regions: no enrichment of reads between two CTCF binding sites (*black dots*) in the *middle panel* compared with the *right panel* with two CTCF binding regions resolved by ChIP-seq. **b** Western blot demonstrates co-immunoprecipitation of CTCF and BORIS complexes: K562 nuclear lysates were immunoprecipitated with IgG, anti-CTCF and anti-BORIS antibodies. Precipitated complexes were probed with CTCF antibodies. The lysates were either untreated (−) or treated (+) with ethidium bromide (*EtBr*) before co-immunoprecipitation. **c**, **d** In situ proximity ligation assay (*ISPLA*) shows the close proximity between CTCF and BORIS proteins in BORIS-positive cells: ovarian cancer cells (OVCAR8) (**c**) and human testis tissue (**d**). **e** A 200-bp ^32^P-labeled probe representing human *TP53* promoter (2xCTS, the palindromic orientation of CTCF motifs shown by *arrows*) was incubated with increasing concentrations of the 11 ZF domain of CTCF (*11ZFs*, *left* and *middle panels*) or *Pichia* recombinant full-length CTCF (*FL CTCF*, *right panel*). The 2xCTS was used in EMSA either as wild type with two CTCF binding sites (*grey boxes*) or as the mutant type with one CTCF mutated site (*red cross*). The model of 11 ZFs and full-length CTCF occupancies are shown by *arrows* and *red dots* (CTCF molecules). **f** A 200-bp ^32^P-labeled probe representing IRF2BP1 3’ untranslated region (UTR) was incubated with nuclear extracts (*n.e.*) from either K562 or NHDF cells. All lanes contain the indicated nuclear extract, except the first lane for K562, where only free probe is present. Nuclear extracts and probe were also incubated with either control mouse IgG (−), antibodies against CTCF or BORIS. The *red* and *blue arrows* point to the super-shifted bands corresponding to CTCF–DNA and BORIS–DNA complexes, respectively. NHDF nuclear extract did not produce a super-shift band with anti-BORIS antibodies. **g** Gene tracks show that exogenous BORIS expression in MCF7 cells (MCF7 + BORIS) recapitulates endogenous BORIS occupancy in OVCAR8 cells. The name of the molecules against which antibodies were directed and the cell lines used in ChIP-seq are shown in the tracks. **h** Heatmap of BORIS (*blue*) occupancy at 50,000 CTCF peaks invariantly mapped in both OVCAR8 and MCF7 + BORIS cells. The tag density was subjected to k-means ranked clustering with two clusters expected. **i** ISPLA in BORIS-negative MCF7 cells stably transfected with either empty vector (*MCF7+EV*) or BORIS (*MCF7+BORIS*). The specific positive ISPLA signal (*red*) is present only in MCF7 cells transfected with BORIS-expressing vector

The short linear distances between the clustered CTCF motifs would likely support protein–protein interactions between the bound partners at these regions in vivo. Indeed, the inability to resolve two closely located CTCF binding sites by ChIP-seq (Fig. 3a) directly supports CTCF and BORIS interaction at 2xCTSes [40]. To test this hypothesis, we performed co-immunoprecipitation experiments and showed that CTCF and BORIS were associated with each other in K562 cell nuclear extracts in the presence and absence of DNA (Fig. 3b). We also performed an in situ proximity ligation assay (ISPLA) in BORIS-positive ovarian cancer cells (OVCAR8) and human testis tissues, confirming that CTCF and BORIS proteins are co-localized in a chromatin context of both cancer and germ cells where the two proteins are co-expressed (Fig. 3c, d). We also observed increased co-occupancy of DNA by CTCF with the wild-type probe (*TP53* promoter) compared with a probe of the same length but with one of the two CTCF sites mutated (Fig. 3e). However, EMSA with increasing amounts of full-length CTCF (Fig. 3e) showed not only increased occupancy of both sites but also the appearance of a third, slower migrating band that likely corresponds to a higher-order CTCF–DNA complex. Furthermore, EMSAs with K562 nuclear extracts demonstrated that the DNA–protein complexes were generally completely super-shifted with both anti-CTCF and anti-BORIS antibodies (Fig. 3f; Fig. S5c in Additional file 5), indicating preferential binding kinetics for heteromeric versus monomeric complexes. Thus, based on several complementary approaches, we can conclude that CTCF and BORIS interact directly at 2xCTSes.

The pattern of CTCF and BORIS occupancy across different cancer cell lines raises the question of whether CTCF and BORIS heterodimerization is preprogrammed in the genome sequence by the 2xCTSes. To test this idea, we induced ectopic BORIS expression in the BORIS-negative breast cancer cell line MCF7 (Fig. 3g–i). The pattern of exogenous BORIS occupancy completely recapitulated the binding profile of endogenous BORIS expression in OVCAR8 cells (Fig. 3g, h). Replacement of CTCF homodimers by CTCF and BORIS heterodimers was further confirmed by ISPLA where the positive signal was detected only in nuclei of MCF7 cells with exogenous BORIS expression (Fig. 3i). Thus, evidently, CTCF and BORIS heterodimeric complexes are not only common in cancer cells of different origins, but can be formed upon induction of BORIS expression in BORIS-negative cells.

### Clustered CTCF binding sites are strongly enriched at active promoters and enhancers in cancer cells

**Fig. 4:**
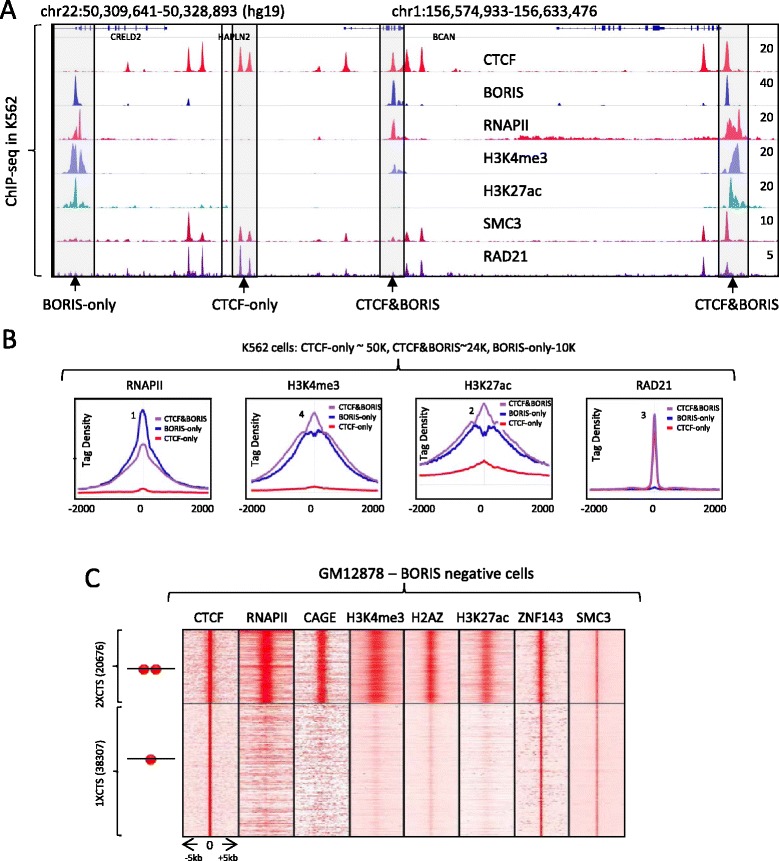
1xCTSes and 2xCTSes are associated with different epigenetic landscapes in both BORIS-positive (K562) and BORIS-negative (GM12878) cells. **a** Gene tracks show the association of CTCF and BORIS bound regions in K562 cells with multiple ENCODE ChIP-seq data. The tracks are labeled with the names of molecules against which antibodies were directed for ChIP-seq. The *gray frames* highlight CTCF-only, CTCF&BORIS and BORIS-only bound regions. **b** Average tag density (tags/10 million) of multiple factors mapped by ChIP-seq in K562 cells (ENCODE data) across BORIS-only (*blue*), CTCF-only (*red*) and CTCF&BORIS (*purple*) bound regions mapped in K562 cells. The data were normalized to the number of mapped reads and binding regions. The names of factors used in ChIP-seq are labeled on the top of each plot. *RNAPII* RNA polymerase II. **c** Heatmap demonstrates the difference between 1xCTSes and 2xCTSes with respect to the enrichment of RNAPII, CAGEs, H3K4me3, H2AZ, H3K27ac, ZNF143, and SMC3 ChIP-seq tag density at two types of CTCF binding classes in BORIS-negative cells (GM12878)

To investigate the functional relevance of clustered CTCF binding sites, we analyzed chromatin organization and the epigenetic landscape of cancer cells at these regions. Comparison of the 2xCTSes (BORIS bound regions: CTCF&BORIS and BORIS-only) identified in K562 cells with the set of data generated by ENCODE showed that most 2xCTSes were specifically associated with active enhancers and promoters, in stark contrast to 1xCTSes (CTCF-only bound regions) (Fig. 4a, b; Additional file 7). About 87 % (15,305) of all active promoters (17,656) in K562 cells, marked by enrichment of the transcription initiating form of RNA polymerase II and the active histone mark H3K4me3, had BORIS occupancy within 4 kb of transcription start sites (TSSs) with the preferential co-occupancy by both CTCF and BORIS proteins (Fig. S7a in Additional file 7). In contrast, only 4 % (666) of these promoters contained single CTCF sites.

Similarly, 50 % (8714) of all active enhancers (17,798) in K562 cells, as defined by H3 histone K27 acetylation and p300 enrichment (histone acetyltransferase), coincided with BORIS bound regions (Fig. S7a in Additional file 7). Recently, a subclass of enhancers, called super enhancers, was shown to orchestrate cell-specific transcription to determine cell identity [41]. More than 76 % (563) of super enhancers identified in K562 overlapped with BORIS bound regions (Fig. S7b in Additional file 7).

Upon further analysis, we established that clustered CTCF binding sites showed dramatically different chromatin features compared with 1xCTSes (Fig. 4a, b; Additional file 7). Factors involved in active transcription, such as RNA polymerase II (RNAPII), TAF1, YY1, TBP, MYC, HMGN3, and PHF8 were highly (80–99.7 %) enriched at CTCF&BORIS bound regions but depleted (0–10 %) from CTCF-only bound regions (Fig. S7c–f in Additional file 7). In contrast, the cohesin complex was enriched at all CTCF bound regions, independent of BORIS occupancy (Fig. 4b; Fig. S7c–f in Additional file 7). Remarkably, RAD21 and SMC3 were depleted from BORIS-only bound regions, suggesting that BORIS was not able to recruit the cohesin complex without CTCF (Fig. 4b; Fig. S7c, d, f in Additional file 7). Thus, BORIS-only and CTCF&BORIS bound regions (2xCTSes) are dramatically different from CTCF-only bound regions (1xCTSes) with respect to their genomic distribution (Fig. S7g in Additional file 7) and co-localization with protein partners, levels, and landmarks of transcription, and the epigenetic landscape of K562 cells (Fig. 4a, b; Additional file 7).

To determine whether this difference stems from the presence of BORIS at 2xCTSes or whether 2xCTSes are themselves inherently functionally different from 1xCTSes, we compared CTCF ChIP-seq data for BORIS-negative (GM12878, lymphoblastoid cells, ENCODE data) and BORIS-positive (K562) cells. The genomic regions invariantly occupied by CTCF in both K562 and GM12878 cells were then separated into two groups (1xCTSes and 2xCTSes) based on BORIS occupancy in K562 cells. The CTCF ChIP-seq tag density at the two classes of CTCF bound regions in GM12878 cells demonstrated significantly higher CTCF occupancy at the 2xCTSes compared with 1xCTSes, in agreement with double CTCF occupancy at 2xCTSes in BORIS-negative cells (Fig. S8a in Additional file 8). The double occupancy for CTCF was also confirmed by DNaseI footprints in BORIS-negative NHDFs (Fig. 2d; Additional file 6). Similarly to K562 cells, 2xCTSes but not 1xCTSes were highly enriched with RNAPII, CAGEs, and active histone marks (H3K4me3, H2AZ, and H3K27ac) (Fig. 4c; Fig. S8a, b in Additional file 8), suggesting that 2xCTSes were preferentially associated with active promoters and enhancers in BORIS-negative cells as well as in BORIS-positive cells (K562). Thus, 2xCTSes are functionally specialized in the epigenome, regardless of their occupancy by CTCF and/or BORIS.

### CTCF and BORIS interactions are involved in the transcriptional program of germ cells

**Fig. 5:**
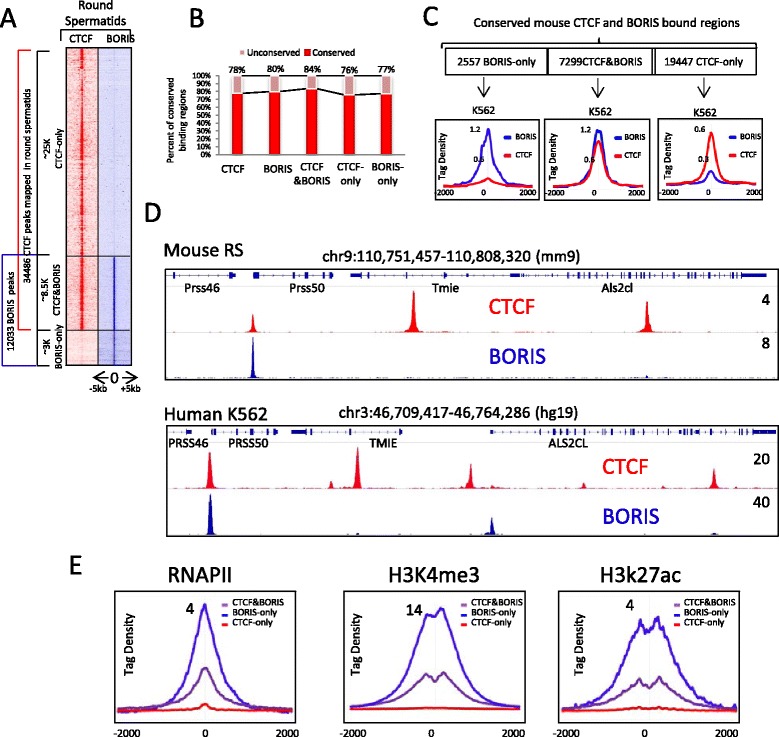
Genomic profiling of CTCF and BORIS occupancy in mouse germ cells. **a** Overlapping of CTCF (*red*) and BORIS (*blue*) genomic occupancy in mouse round spermatids. **b** Histogram indicates the percentage of conserved mouse CTCF and BORIS bound regions in the human genome. **c** Average tag density of CTCF (*red*) and BORIS (*blue*) occupancy in K562 cells across the conserved mouse binding regions from (**b**). **d** Gene tracks of CTCF and BORIS occupancy at the *Tsp50*/*TSP50* (*Prss50*/*PRSS50*) gene in mouse (round spermatids) and human (K562) cells. **e** Average tag density (tags/10 million) of RNAPII, H3K4me3, and H3K27ac mapped by ChIP-seq in mouse testis (ENCODE data) across BORIS-only (*blue*), CTCF-only (*red*) and CTCF&BORIS (*purple*) bound regions mapped in mouse round spermatids. The data were normalized to the number of mapped reads and binding regions

The genomic binding patterns of CTCF and BORIS in cancer cells suggest that the heterodimerization between these two proteins could also be characteristic for germ cells. CTCF and BORIS are co-expressed during spermatogenesis, with BORIS expression being the highest in haploid round spermatids compared with other germ cells [27]. To examine the interplay between BORIS and CTCF in the native context, we performed ChIP-seq to determine the occupancy of the two proteins in elutriated mouse round spermatids. Similar to cancer cells, 25 % of CTCF bound regions were co-occupied by BORIS in round spermatids (Fig. 5a). Importantly, a high level of conservation was observed for both CTCF and BORIS bound regions in germ cells (Fig. 5b): 84 %, 76 % and 77 % of CTCF/BORIS, CTCF-only and BORIS-only bound regions, respectively, could be aligned to the human genome. Furthermore, these regions were occupied in the same manner by CTCF and BORIS in K562, OVCAR8 and Delta47 cells (Fig. 5c; Additional file 9). The fact that similar patterns of CTCF and BORIS occupancy were observed for the conserved genomic regions in both human cancer cell lines and mouse germ cells (Fig. 5c, d; Additional file 9) strongly indicates that CTCF and BORIS co-occupancy in cancer cells recapitulates the features of germline chromatin.

As BORIS bound regions (2xCTSes) were highly enriched at the active promoters and enhancers in human cancer cells (Fig. 4), we analyzed the enrichment of RNAPII, H3K27ac, and H3K4me3 mapped in mouse germ cells (mouse testis, ENCODE) in correlation with BORIS-bound regions (CTCF&BORIS and BORIS-only) and CTCF-only bound regions mapped in round spermatids. Similarly to cancer cells (Fig. 5e), all three marks of active transcription were significantly enriched at BORIS-bound regions compared with CTCF-only bound regions, signifying the involvement of 2xCTSes in germline transcription regulation.

### 2xCTSes demonstrate distinct functional properties upon co-binding by CTCF and BORIS or by CTCF homodimers

**Fig. 6:**
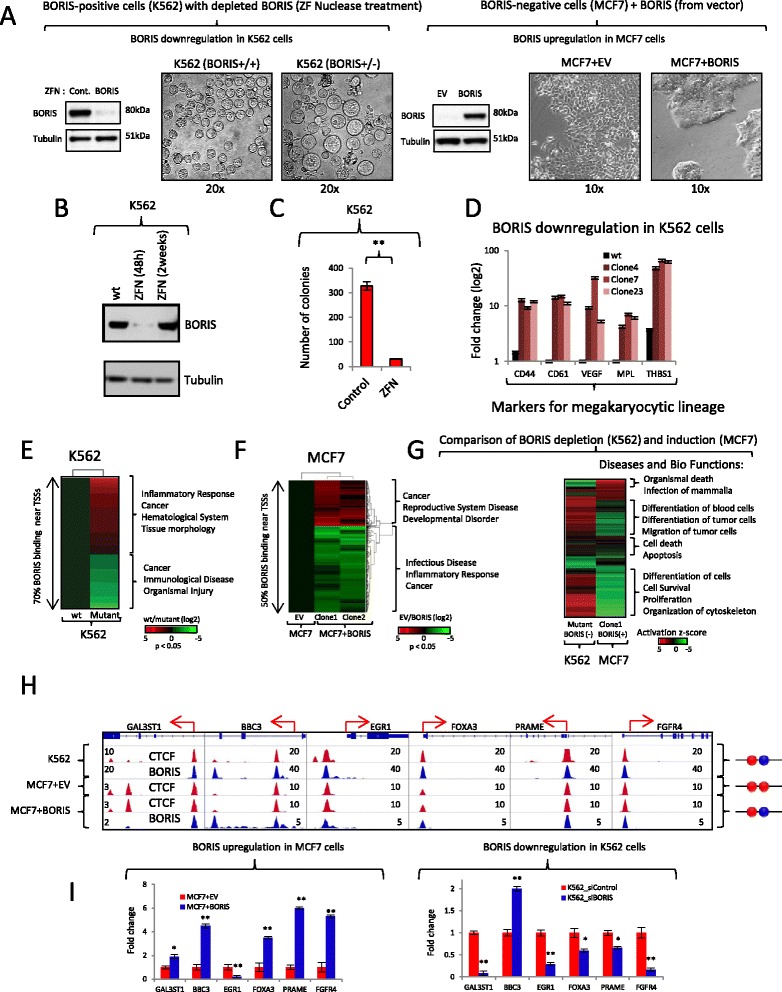
CTCF and BORIS interactions are essential for transcriptional regulation in cancers. **a** Two models are used in the study: K562 cells (*BORIS+/+*) treated with zinc finger nuclease (*ZFN*) to knockout/downregulate the BORIS gene (*BORIS+/−*) and MCF7 cells with stably transfected empty vector (*MCF7+EV*) or BORIS-expressing vector (*MCF7+BORIS*). Upon stable change of BORIS expression (western blot) both types of cells showed phenotypic changes. **b** Western blot analysis of K562 cells (mass culture) upon BORIS depletion by ZFN (before ZFN (*wt*), 48 h, and two weeks after ZFN). **c** Number of K562 single-cell clones growing in soft agar after ZFN transfection (*ZFN*) compared with untransfected cells (*Control*). **d** Expression of megakaryocytic lineage markers in three independent single-cell K562 clones after ZFN treatment compared with wild type (*wt*). **e**, **f** Fold changes (log2) in gene expression in response to BORIS depletion in K562 cells (**e**) and BORIS induction in MCF7 cells (**f**). Two independent single-cell clones of MCF7 cells with stably expressed BORIS were analyzed (**f**). Top associated diseases and bio functions (*p* < 0.0001) are shown for upregulated (*red*) and downregulated (*green*) genes on the right (Ingenuity Pathway Analysis). The genes that change expression in a similar way in both independent MCF7 clones with stably transfected BORIS are shown on the heat map (**f**). **g** Heat map showing the comparison of genes that changed expression upon BORIS depletion (K562 cells) and BORIS induction (MCF7 cells, clone1) in respect to diseases and bio functions (activation z-score). **h** Genome browser view of six CTCF&BORIS target genes. The tracks are labeled with the names of molecules against which antibodies were directed and the cell lines used in ChIP-seq, respectively. The *red arrows* show the beginning and direction of transcription. The schematic occupancy of promoters with either CTCF (*red*) and BORIS (*blue*) heterodimers or CTCF homodimer is shown on the *right*. **i** Expression of six CTCF&BORIS targets from panel (**h**) was analyzed by quantitative PCR in cells with upregulated (*MCF7+BORIS*) and downregulated BORIS (BORIS small interference RNA (*si*) treatment of K562 cells, mass culture) expression. Asterisk (*) represents *p*-value <0.05 and double-asterisk (**) represents *p*-value <0.01 between treated and untreated cells (c, i). Error bars represent standard deviation of the average of triplicate measurements (c, d, i). *TSS* transcription start site

As 2xCTSes were found more frequently within promoters and enhancers in dramatic contrast to 1xCTSes (Figs. 4 and 5), we tested whether binding of both CTCF and BORIS to these regions would have a different impact on transcription compared with CTCF binding alone. We employed two independent experimental systems to modulate the level of BORIS protein without changing the expression of CTCF (Fig. 6a). The introduction of zinc finger nuclease (ZFN), targeting the first coding exon of the BORIS gene, resulted in high efficiency of BORIS knockout 48 h after transfection (Fig. 6a; Fig. S10a, b in Additional file 10). However, K562 cells with mutations in BORIS were eventually depleted with extended culturing, suggesting a requirement of BORIS for cell viability or proliferation (Fig. 6b; Fig. S10b–d in Additional file 10). The latter was a distinct possibility, as it is known that the K562 cell line can be differentiated into either erythroid or megakaryocytic lineages [42]. Consistent with this model, K562 cells treated with ZFN produced significantly fewer colonies compared with untreated cells when plated in soft agar (Fig. 6c; Fig. S10e in Additional file 10). Single-cell clones (50 clones) recovered from soft agar, upon analysis for the efficiency of mutagenesis by CEL-I assay, immunoblotting, and DNA sequencing, all contained a wild-type BORIS allele, with 20 % of the clones also having one allele of the BORIS gene mutated (Fig. S10c, d in Additional file 10). A second round of treatment of mutated clones with ZFN produced a more severe phenotype with indications of megakaryocytic differentiation (Fig. 6a), such as the upregulation of megakaryocytic markers in all mutated single-cell clones with heterozygous BORIS deletion (Fig. 6d; data not shown). A full knockout of the BORIS gene in K562 cells had never been obtained despite multiple attempts (data not shown), indicating that BORIS knockout could be incompatible with the viability of K562 cells. However, we clearly established that BORIS is required for K562 proliferation, as the loss of BORIS led to the differentiation of K562 cells into the megakaryocytic lineage (Fig. 6a, d). Conversely, the differentiation of K562 cells into the megakaryocytic lineage by independent means (i.e., upon phorbol 12*-*myristate 13*-*acetate (PMA) treatment led to the dramatic downregulation of BORIS, signifying BORIS involvement in maintenance of K562 multipotency (Fig. S10f, g in Additional file 10).

To directly gauge the involvement of BORIS in the transcriptional regulation of K562 cells we performed RNA-seq analysis of K562 upon BORIS depletion. Hundreds of genes functionally linked to cancer, inflammatory response, and/or cell differentiation changed their expression levels upon BORIS downregulation in K562 cells (Fig. 6e; Tables S1–S3 in Additional file 11). In particular, the depletion of BORIS in K562 cells resulted in significant change in transcription of 1035 genes (351 downregulated and 684 upregulated, *p* < 0.05; Fig. 6e). The majority of the genes (70 %) that changed transcription had either CTCF&BORIS binding (Fisher’s exact test, *p* = 0.0095, odds ratio = 1.29) or BORIS-only binding (Fisher’s exact test, *p* < 0.0001, odds ratio = 1.3) in the promoter region (±5 kb from TSSs).

In the second experimental system, we ectopically expressed BORIS in the cell line MCF7, which does not normally express this gene (Fig. 6a). Following stable BORIS expression in two independent clones, we observed a dramatic change toward a stem-like phenotype (Fig. 6a). A similar phenotype was described in MCF7 cells upon loss of WISP2 (Wnt-1-induced signaling protein-2) expression [43]. Indeed, upon RNA-seq analysis of MCF7 cells with stable BORIS expression we found a dramatic downregulation of WISP2 in clone 1 and almost complete knockdown of WISP2 in clone 2 (Fig. S10h in Additional file 10). The ectopic expression of BORIS in MCF7 cells resulted in the deregulation of 2022 (1334 downregulated and 688 upregulated, *p* < 0.05) and 2366 (1191 downregulated and 1175 upregulated, *p* < 0.05) genes in clones 1 and 2, respectively (Fig. 6f). In line with the direct involvement of BORIS in gene regulation in *cis*, the majority of genes (55 % in clone 1, 67 % in clone 2 that changed transcription levels upon induced BORIS expression showed either CTCF&BORIS binding (Fisher’s exact test; clone 1, *p* < 0.0001, odds ratio = 1.52; clone 2, *p* < 0.0001, odds ratio = 1.69) or BORIS-only binding (Fisher’s exact test; clone 1, *p* < 0.0001, odds ratio = 1.47; clone 2, *p* < 0.0001, odds ratio = 1.47) in the promoter region (±5 kb from TSSs). The main pathways concordantly up- or downregulated in both independent clones were significantly associated with cancer, inflammatory response, and reproductive system disease (Fig. 6f; Tables S4–S6 in Additional file 11).

The detailed comparison of cells with depleted or induced BORIS expression demonstrated the changes in transcriptional outcomes that strongly correlated with BORIS levels in both systems (Fig. 6e–g; Tables S7 and S8 in Additional file 11). For example, inflammation pathways were downregulated with induced BORIS expression in MCF7 cells while being upregulated upon BORIS downregulation in K562 (Fig. 6e–g; Fig. S10i in Additional file 10; Tables S7 and S8 in Additional file 11). Furthermore, the genes involved in megakaryocytic lineage differentiation were highly upregulated in BORIS-depleted K562 cells (Fig. 6a, e), while the genes involved in the differentiation of cells were downregulated in MCF7 cells upon BORIS expression (Tables S7 and S8 in Additional file 11).

Comparing the cell lines with differential occupancy at 2xCTSes also implicated the direct involvement of BORIS at these regions in transcriptional regulation. As illustrated in Fig. S10k, l in Additional file 10, the genes with 2xCTSes where CTCF occupancy (NHDF) was replaced by CTCF and BORIS co-occupancy (K562, OVCAR8, Delta47) displayed distinct modes of transcriptional regulation. For example, the testis-specific promoter of the *GAL3ST1* gene was silenced when occupied by CTCF alone in most BORIS-negative cell lines, but it was activated in BORIS-positive cells (germ and cancer) when co-occupied by CTCF and BORIS (Fig. S10k, l in Additional file 10). Furthermore, the experimental upregulation or depletion of BORIS led to an increase or decrease of *GAL3ST1* expression, respectively (Fig. 6h, i). Similarly to *GAL3ST1*, *PRAME* and *FOXA3* genes were also silent under CTCF occupancy, but were activated upon CTCF and BORIS co-binding in both cancer and germ cells (Fig. S10k, l in Additional file 10). An opposite occupancy-specific effect was observed for the *EGR1* promoter, which was highly active under CTCF homodimer occupancy in MCF7 cells but was dramatically repressed upon ectopic BORIS expression (Fig. 6h, i). Two more examples, *BBC3* and *FGFR4*, showed dissimilar transcriptional outcomes depending on 2xCTS occupancy by CTCF and BORIS (Fig. 6h, i). Thus, these analyses showed that CTCF&BORIS co-regulation of corresponding genes is of critical importance for the transcriptional program of both germ and cancer cells, and represents a functionally distinct mode of transcription control compared with CTCF homodimer bound at the same regions.

We also found some revealing examples of CTCF&BORIS controlled genes that changed their expression in the same way upon both BORIS induction and depletion: *BBC3* was upregulated and *EGR1* downregulated (Fig. 6h, i). This could be explained either by an indirect effect of BORIS balance or by BORIS regulation implemented through long-range chromatin interactions. The latter is an intriguing option, as the potential role of BORIS in three-dimensional folding of chromatin had never been >possibility we cross-referenced our ChIP-seq data with a published RNAPII ChIA-PET study where 30,000 K562-specific loops were mapped in BORIS-positive cells (K562) versus BORIS-negative cells (MCF7) [44]. CTCF&BORIS bound regions were notably overrepresented (65 %) at the anchors of K562-specific loops (Fig. S11a in Additional file 12). More importantly, the same regions were occupied by CTCF and RNAPII in MCF7 cells, but the long-range interactions were different (Fig. S11b, c in Additional file 12). Thus, BORIS may re-wire the long-range chromatin interactions mediated by CTCF in BORIS-negative cells through CTCF and BORIS heterodimerization in BORIS-positive cells (the proposed model is presented in Fig. S11d in Additional file 12).

### Clustered CTCF motifs are associated with specific chromatin architecture in human and mouse sperm

**Fig. 7:**
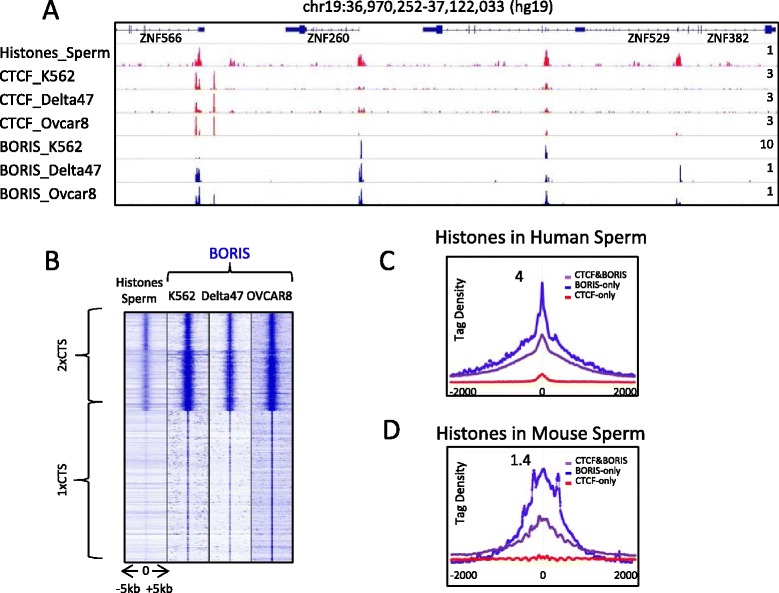
BORIS bound regions correlate with histone retention in human and mouse sperm. **a** Gene tracks show the distribution of histones retained in human sperm in association with CTCF and BORIS bound regions, mapped in K562, Delta47 and OVCAR8 cell lines. The name of the molecules against which antibodies were directed and the cell lines used in ChIP-seq are shown on the left. **b** Heatmap indicates the enrichment of histones retained in human sperm at 2xCTS regions (invariant BORIS binding in K562, OVCAR8, and Delta47). **c, d** Average tag density (tags/10 million) of histones retained in human (**c**) and mouse (**d**) sperm across BORIS-only (*blue*), CTCF-only (*red*) and CTCF&BORIS (*purple*) bound regions mapped in K562 cells (**c**) and mouse round spermatids (**d**). The data were normalized to the number of mapped reads and binding regions

As shown here, CTCF and BORIS were co-localized in vivo in postmeiotic haploid round spermatids (Fig. 5). Round spermatids undergo an extensive chromatin remodeling process during their final differentiation into mature sperm. During these processes DNA is packaged into a highly condensed state in which the somatic histones are replaced by protamines. However, a small proportion of histones remains associated with DNA and there are several reports suggesting CTCF association with these regions [45–48]. In order to understand the biological function of 2xCTSes during male germ cell development, we compared the two classes of CTCF binding regions with the genomic loci that escape protamine occupancy in mature human and mouse sperm. For that we used a published data set of MNase footprints mapped in human and mouse sperm [49, 50]. The overlap between our set of data for CTCF and BORIS occupancy in cancers with the regions of histone retained in human sperm showed the specific enrichment of histones at 2xCTSes, but not at 1xCTSes (Fig. 7a, b). To extend this observation further, we analyzed the enrichment of histones retained in sperm at two classes of CTCF binding regions: 2xCTSes (CTCF&BORIS, BORIS-only) and 1xCTSes (CTCF-only). We found that in both human and mouse sperm, the histones were indeed specifically retained at BORIS-bound regions (Fig. 7c, d), but not at CTCF-only bound regions. These results suggest that occupancy of BORIS at 2xCTSes may be implicated in chromatin remodeling during the differentiation of round spermatids, where BORIS is highly expressed, and may mark the regions where the histones have to be retained to label genes, promoters, and enhancers destined for early expression in the developing embryo.

## Discussion

ChIP-seq analysis of several BORIS-expressing cancer cell lines established that the pattern of BORIS binding is similar across cell lines of independent origin and thus likely reflects an underlying “encoding” of the binding regions for their propensity to bind BORIS (Fig. 1; Additional file 1). Upon further analysis, we uncovered that this encoding largely reflects the ability of these regions to be co-bound by CTCF and BORIS and/or by BORIS or CTCF homodimers. Such regions, mapped by ChIP-seq as single peaks, encompass two or more closely spaced CTCF binding DNA sequences/motifs (used here interchangeably as 2xCTS or clustered CTSs). We further showed that structural properties of 2xCTSes and single CTCF sites (1xCTSes) correlate with functional differences: 2xCTSes are preferentially associated with promoters and enhancers, confer differential modes of transcriptional regulation depending on the occupancy by CTCF and/or BORIS, and correlate with regions that retain histones during spermatogenesis.

### Genome-wide analysis of BORIS distribution reveals the inherent structural dichotomy between clustered CTCF binding sites and single CTCF binding sites

Our present analysis of BORIS distribution in chromatin of cancer cells (Fig. 1), in addition to providing a wealth of information on the involvement of BORIS in transcriptional regulation and chromatin organization, inadvertently revealed inherent differentiation of CTSes. Approximately one-third of CTCF binding regions identified by ChIP-seq as single peaks encompassed at least two closely spaced CTSes (Fig. 2). The 2xCTSes were predisposed to bind BORIS in cooperative fashion with CTCF (CTCF&BORIS), as well as comprised the majority of BORIS-only sites (Fig. 2; Additional files 1, 2, and 3). Notwithstanding that their genome wide-distribution was revealed by the present BORIS analysis, the emergence of 2xCTSes evidently predated the appearance of BORIS in evolution. The existence of these clustered sites was demonstrated in our previous work on EMSA mapping of CTCF binding [32, 51]. With longer (150–200 bp) EMSA probes, we repeatedly detected either one or two DNA–protein complexes with the 11 ZF DNA binding domain of CTCF ([32, 51, 52] and unpublished observation). Further analyses of a subset of CTCF targets uncovered the corresponding presence of either one or two CTSes within the probes [32, 51]. For example, the Fab-8 *Drosophila* chromatin insulator was found to encompass two closely spaced CTSes [51], while the testis-specific promoter of *TSP50* was shown to include two adjacent CTSes, conserved between mouse and human [32]. The published examples of two closely spaced CTSes, as we now know, include alternative BORIS promoters [34], mouse KvDMR1 imprinting locus [52], BAX promoter [53], enhancers of the murine Igh locus [54], and others [55]. The principal biological significance of clustered CTSes can be seen in their evolutionary conservation. For example, the two adjacent CTSes in the *PRSS50*/*TSP50* promoter are highly conserved, and the CTCF-binding motifs are arranged similarly in human and mouse promoters (Fig. 2a). In the *TP53*/*Tsp53* promoter, the two CTCF sites are arranged in opposite directions, yet in a very similar way in both human and mouse (Fig. S4b in Additional file 4).

The structural difference of clustered CTSes from 1xCTSes is based on the number, i.e., two or more, of ZF-bound DNA motifs. The existence of clustered CTSes is also likely constrained by the requirement for close spacing of DNA motifs, as suggested by their single-peak appearance in ChIP-seq (Fig. 3a). The widespread genomic occurrence of clustered sites was not revealed until the present work, largely due to the insufficient resolution of ChIP-seq experiments and the perception in published studies that all CTCF ChIP-seq peaks comprise a single CTCF motif [56].

In this work, we present evidence that 1xCTSes are preferentially occupied by CTCF only and contain a single CTCF binding motif, while clustered CTSes, which enclose two or more CTCF-binding motifs, are preferentially occupied by larger/multimeric complexes, including CTCF&CTCF, CTCF&BORIS, or BORIS&BORIS (Fig. 2; Additional files 3, 4, and 5). This model is supported by the demonstration of the presence of at least two CTCF motifs and at least two DNaseI footprints in the regions with 2xCTSes (Fig. 2d; Additional file 6), as well as by the conservation of two juxtaposed CTCF motifs according to phastCons score (Additional file 6). Additionally, we showed that clustered CTSes predispose the physical interactions of CTCF and BORIS (Fig. 3). Further, results from EMSA (Fig. 3f; Fig. S5c in Additional file 5), ChIP-Re-ChIP (Fig. 1i; Fig. S2b in Additional file 2), co-immunoprecipitation (Fig. 3b), and ISPLA in both cancer and germ cells (Fig. 3c, d, i) support the co-occupancy of 2xCTSes by CTCF and BORIS. The discovery of 2xCTSes adds an additional layer of complexity to the versatility of CTCF, and likely BORIS, as multifunctional chromatin factors. The CTCF, a multifunctional protein itself [12], employs variable combinations of 11 ZFs to bind a wide range of DNA sequences, which form an extensive array of motifs [13, 56]. In that context, the clustered CTSes, with variable spacing, orientation, and the number of binding sites that are bound by several CTCF and/or BORIS molecules, would confer a substantially higher degree of versatility to the regulatory potential of CTCF and/or BORIS. These chromatin regions likely have a higher degree of combinatorial usage of ZFs, stronger binding, more cooperative protein–protein interaction, a wider spectrum of protein partners, and likely more selective interactions with other anchors of chromatin loops compared with single sites.

### 2xCTSes as a transcriptional platform modulated by BORIS

The discovery of clustered CTSes enables us, for the first time, to address the long-standing question of how CTCF can serve in the context of the same nucleus as a bona fide transcription factor, while maintaining a substantial presence at putative insulator/boundary sites that bear no indications of transcriptional activity [1]. Indeed, only 10–20 % of all CTCF binding regions are located in promoter regions in any given cell type [14, 57], while the rest of the CTSes are not associated with TSSs. The obvious candidates for the determinants of such distinct functional roles would be DNA sequences themselves and/or differential identity of chromatin at these two types of sites. Here, we present genome-wide evidence that DNA sequences underlying the two types of CTSes are structurally different, i.e., that not all CTCF bound regions are equal. It is exemplified by the finding that only clustered CTSes, but not 1xCTSes, were associated with active promoters and enhancers in both cancer and germ cells (Figs. 4 and 5). Thus, one could hypothesize that the role of CTCF, and likely BORIS, as transcription factors is implemented through 2xCTSes. As with other transcription factors, that role could be further modulated by the interactions with additional proteins [58–63]. Furthermore, in case of CTCF and/or BORIS serving in a transcription factor capacity, their potential to regulate gene expression may also involve a formation of chromatin loops (Additional file 12).

The question that remains open is what mechanism facilitates BORIS replacement of CTCF at clustered CTSes to form CTCF&BORIS and BORIS-only bound regions. The originally proposed model for BORIS’ “invasion” of CTSes upon its expression in germline and cancers was that BORIS simply outcompetes CTCF for binding at some sites [27, 30]. However, based on the present genome-wide study, it is more likely that BORIS outcompetes CTCF from one out of two closely spaced binding sites at the majority of 2xCTSes, therefore replacing CTCF homodimer with a more stable CTCF&BORIS heterodimer, and that BORIS-only bound regions represent the next step in the full replacement of CTCF by BORIS. The outcome of CTCF and BORIS homodimer and heterodimer occupancy at 2xCTSes is different with respect to transcriptional regulation and, possibly, transcriptional loop formation (Fig. 6; Additional files 10 and 12). In contrast to clustered CTSes, the 1xCTSes evidently are preferentially located intergenically and associated with genomic regions devoid of hallmarks of active transcription (Fig. 4; Additional files 7 and 8). These characteristics may be consistent with chromatin barrier/insulator elements.

### BORIS binding to DNA and its putative regulatory function in cancer cells is causally related to CTCF&BORIS and BORIS-only bound regions in the germline

CTCF is ubiquitously expressed in all types of cells, while BORIS expression is normally restricted to germ cells and could be aberrantly activated in cancers [27, 35, 38]. The aberrant activation of cancer testis genes, especially global regulators such as BORIS, could be a substantial component of reprogramming normal somatic cells into malignant ones. Here we demonstrate that the core binding pattern of BORIS distribution in three independent cell lines that tolerate high levels of endogenous BORIS is probably rooted in the normal function of BORIS itself and its interaction with CTCF in the germline. This conclusion is primarily based on our finding that the genome-wide occupancy of 1xCTSes and 2xCTSes by CTCF and BORIS in cancer cells largely recapitulates their binding profile in germ cells, at least for the subset of binding regions that are conserved between mice and humans (Fig. 5; Additional file 9).

As clustered CTSes were strongly associated with transcription (Fig. 4) and enriched for active enhancer and promoter epigenetic marks (Figs. 4 and 5e), it was logical to hypothesize that BORIS binding and/or BORIS heterodimerization with CTCF at clustered CTSes may initiate or at least predispose cancer cells for the actual execution of a germline-like transcription program. Indeed, upon examining the actual effect of BORIS dosage on the transcription of genes associated with clustered CTSes, we found that the substitution of CTCF homodimer by CTCF and BORIS heterodimer at the 2xCTSes is required for the expression of some testis-specific genes (Fig. 6; Fig. S10k, l in Additional file 10). Furthermore, using two independent systems modulating BORIS levels in opposing directions, we demonstrated that the transcription of genes regulated by BORIS is actually dependent on BORIS dosage for most cases, suggesting the direct mode of regulation (Fig. 6). The mechanism of this predisposition of cells aberrantly expressing BORIS to the germline-specific transcriptional configuration of the epigenome and, to some degree, transcriptome (Fig. 6) is likely implemented via chromatin loops specific for BORIS-bound clustered sites (Additional file 12). This finding opens a new chapter in our understanding of CTCF-mediated chromatin loop organization genome-wide, as it assigns this crucial CTCF function to a specific subset of CTSes, namely clustered CTSes. The actual levels of transcriptional activation of germline-specific genes controlled by clustered CTSes bound by BORIS in cancer cells can be, of course, additionally dependent on other factors known to be deregulated in cancers, such as DNA methylation and the expression of other tissue-specific transcription factors. For example, while the *TSP50* gene is controlled by the clustered CTS bound by CTCF and BORIS in both human and mouse germ cells (Figs. 2a and 5d), its activation required the full demethylation of the promoter region [32].

### A putative novel function of 2xCTSes in normal postmeiotic germ cells

Understanding the functional role of BORIS in cancer is hardly possible without learning more about its normal function. While the role of BORIS as transcriptional activator of spermatogenesis-specific genes has been established [29, 30, 32], there are additional observations that require more extensive analyses. One such phenomenon is the role of BORIS in postmeiotic cells, where BORIS expression is highest, based on mRNA levels [29]. However, the previous assessment of green fluorescent protein-BORIS protein distribution in transgenic mice indicated that, while its level is high in spermatogonia and preleptotene spermatocytes, it is below recordable levels in postmeiotic cells [30]. The reliable detection of untagged/native BORIS protein is particularly challenging due to the lack of reliable commercial antibodies, which is exemplified by the false positive detection of BORIS expression in somatic tissues [64]. Our results with newly generated anti-mouse BORIS polyclonal antibodies confirmed the expression of BORIS in round spermatids (Fig. 5a), where a wave of postmeiotic transcription and chromatin remodeling takes place to produce mature spermatozoa. Concomitantly, chromatin becomes more condensed, and histones are replaced by protamines, with only 5–10 % of the sperm’s epigenome still packaged with histones [45–50, 65–69]. The regions that retained histones have been reported to be essential for the expression of genes related to early zygotic activation [47, 48, 50]. Moreover, the presence of CTCF protein in mouse and human sperm has been reported previously [70]. In addition, several reports have shown the presence of the CTCF motif and correlation with CTCF bound regions in different cells lines with MNase footprints released by micrococcal nuclease digestion in human and mouse sperm [45–47]. Although our results concur, we found that not all CTCF binding regions are correlated with histone retention in sperm, but 2xCTSes specifically. Those regions are either co-occupied by BORIS only or by both CTCF and BORIS in round spermatids and, likely, in sperm. Those regions also could correlate with testis-specific histones that escape histone-to-protamine replacement in mature sperm [71]. These results raise the intriguing possibility that BORIS plays a regulatory role in sperm genome organization and gene expression during early development. However, data produced by us and others do not show direct binding of CTCF and BORIS to the sperm genome. Future studies with ChIP-seq data of BORIS and CTCF in human sperm will be needed to analyze if the presence of both proteins may play a role in the regulation of the gene expression on zygotic gene activation and whether both proteins bound to sperm chromatin are important for gene expression during development.

## Conclusions

Our study provides the first comprehensive analysis of CTCF and BORIS occupancy in cancer and germ cells, where the two paralogous proteins are co-expressed. In this study we found that BORIS binds to approximately one-third of CTCF bound regions, representing clustered CTCF binding sites, while the remaining two-thirds consist of single CTCF binding sites and are occupied by CTCF alone in vivo. Thus, the pattern of BORIS binding uncovered two classes of CTCF binding regions that are preprogrammed and evolutionarily conserved in DNA sequence. Our study challenges the perception in the current literature that all CTCF sites are equal and characterized by a single CTCF motif. The clustered CTCF binding sites constrain CTCF to form homodimers in normal somatic cells and heterodimers with BORIS in germ and cancer cells expressing BORIS. The drastic enrichment of clustered CTCF binding sites at active promoters and enhancers confirms their functional significance in transcription. In male germ cell development, clustered CTCF binding sites are associated with a unique chromatin composition in sperm, likely presetting these regions for zygotic gene activation.

## Materials and methods

### Cell culture

K562, Delta47, OVCAR8, NHDF, and MCF7 cell lines were grown in Dulbecco's modified Eagle medium (DMEM) supplemented with 10 % fetal calf serum and penicillin-streptomycin. MCF7 cells were transfected using the Cell Line Nucleofector® Kit V (program P-020; Lonza Group Ltd) and the vector (pCpGvitro-hygro, InvivoGen) encoding either LacZ (empty vector) or the open reading frame (ORF) of BORIS. After 3 weeks of hygromycin selection (150 mg/l), several single-cell clones stably growing under antibiotic selection were selected and analyzed by western blot for the presence of BORIS exogenous expression (data not shown). To knockdown BORIS expression, K562 cells were transfected using Cell Line Nucleofector® Kit V (program T-016; Lonza Group Ltd) with either small interfering RNA (siRNA) control or with BORIS SMART pool siRNAs (Dharmacon, Inc.) or with ZFNs, targeting the first coding exon of the BORIS gene. ZFNs targeting the BORIS gene were designed and validated by CompoZr® Custom ZFN Service (Sigma-Aldrich Life Science, St Louis, MO, USA). Capped ZFN mRNAs were produced from linearized plasmid DNA by in vitro transcription with MessageMAX™ T7 ARCACapped Message Transcription Kit and purified by MEGAclear™ Kit (Sigma-Aldrich). The two ZFN mRNAs were combined in equal amounts for gene knockout and delivered to the cells by transfection. As a control we used a single ZFN mRNA. ZFNs were validated for genome editing activity by transient transfection into K562 cells and measurement via the Surveyor Nuclease Assay for endogenous activity. Round spermatids were purified by centrifugal elutriation [72] followed by flow cytometry sorting of cells stained with Vybrant DyeCycle Green (Invitrogen, Carlsbad, CA, USA) to obtain cell fractions with high purity. Briefly, decapsulated testis were treated with collagenase followed by treatment with trypsin, and the dissociated cells were used for centrifugal elutriation. Partially purified spermatocytes and round spermatid fractions were incubated with 10 μM Vybrant DyeCycle Green for 30 min at 32 °C followed by 4,6-diamidino-2-phenylindole (DAPI) staining. Cells were then sorted on a FACSAria (Becton Dickinson) to purify spermatocytes and round spermatids. DAPI-positive dead cells were eliminated. The purity of cells was confirmed by flow cytometry analysis of DNA content. All animal experiments were performed at the NIH/Bethesda, in compliance with the guidelines of the Institutional Animal Care and Use Committee of the National Institute of Allergy and Infectious Diseases (NIAID).

### ChIP sequencing

For ChIP-seq 10^8^ asynchronously growing cells were crosslinked with 1 % formaldehyde for 10 min at room temperature, followed by quenching with 125 mM glycine for 10 min, washed twice with 1× phosphate buffered saline (PBS), and resuspended in chromatin immunoprecipitation (ChIP) lysis buffer (150 mM NaCl, 1 % Triton X‐100, 0.1 % SDS, 20 mM Tris–HCl pH8.0, 2 mM EDTA). Chromatin was sheared to an average length of 200–500 bp using a Bioruptor sonicator. After overnight incubation with DiaMag magnetic beads (Diagenode, Inc.) and CTCF or BORIS monoclonal or polyclonal antibodies (characterized and described by us [29, 32, 39]), precipitated chromatin was then washed, de-crosslinked, and digested with proteinase K. The resulting DNA was purified using phenol/chloroform/isoamyl alcohol. DNA concentration was assessed with a Quant‐it PicoGreen dsDNA kit (Invitrogen) and 5–10 ng was used to generate sequencing libraries. ChIP DNA was amplified using a TruSeq ChIP Sample Preparation Kit (Illumina, Inc., USA). Briefly, the immunoprecipitated material was end-repaired, A-tailed, ligated to the sequencing adapters, amplified by 15 cycles of PCR, and size selected (200–400 bp) followed by single end sequencing on an Illumina Genome Analyzer according to the manufacturer’s recommendations.

### ChIP-Re-ChIP

Chromatin was prepared as for ChIP-seq as described above. First, chromatin was immunoprecipitated using BORIS monoclonal antibodies, which were chemically crosslinked to magnetic beads using crosslinking buffer (0.2 M triethanolamine pH 8.2, 20 mM DMP), 30 min at room temperature. After overnight incubation with crosslinked antibodies, the chromatin was washed and eluted twice using elution buffer (0.1 M glycine-HCl pH 2.5). Eluted chromatin was neutralized using 1 M Tris (pH 8) and used for the second round of ChIP with CTCF monoclonal antibodies following the standard ChIP protocol as described above. The sequencing libraries were prepared as described above.

### Bioinformatic analysis of ChIP-seq data

Sequences generated by the Illumina genome analyzer (36 and 50 bp reads) were aligned against either the human (build hg19) or mouse (build mm9) genome using the Bowtie program [73]. The alignment was performed with default parameters except the sequence tags that mapped to more than one location in the genome were excluded from the analysis using the –m1 option. Peaks were called using Model-based Analysis for ChIP-seq (MACS) [74] using default parameters. After MACS, we applied the Peak Splitter algorithm (part of MACS) to call sub-peaks and summits of peaks and improve peak resolution*.* The ChIP-seq data were visualized using the Integrative Genomics Viewer (IGV) [75]. The peak overlaps between CTCF and BORIS ChIP-seq data sets were determined with BedTools Suite [76]. We defined peaks as overlapping if at least 1 bp of reciprocal peaks intersect (CTCF&BORIS); the remaining peaks were defined as non-overlapping (CTCF-only and BORIS-only). The normalized tag density profiles were generated using the BedTools coverage option from the BedTools Suite, normalized to the number of mapped reads, and plotted by Microsoft Excel. The heatmaps were generated using the seqMINER 1.3.3 platform [77]. We used either k-means ranked or linear method for clustering normalization. The summits of either CTCF or BORIS peaks were extended ±5 kb. seqMINER was also used to generate the average profiles of read density for different clusters. Position weight matrices for CTCF and BORIS bound regions were searched using Multiple EM for Motif Elicitation (MEME) software [78]. The sequences under the summit of either CTCF or BORIS peaks extended 100 bp upstream and downstream were used for motif discovery. We ran MEME with parameters (−mod oops -revcomp -w 20) to identify 20-bp-long motifs considering both DNA strands. To analyze the occurrence of CTCF motifs in the sequences occupied by either CTCF or BORIS or both proteins at the same time, we used FIMO software (MEME suite) with default parameters. The position weight matrices found for CTCF binding regions by MEME were used for FIMO software. Each CTCF motif occurrence had a *p* value < 0.0001 in the sequences of 200 bp around the summit of either CTCF (CTCF-only, CTCF&BORIS bound regions) or BORIS (BORIS-only bound regions) peaks. For evolutionary conservation analysis, all pre-computed phastCons scores were obtained from the University of California, Santa Cruz (UCSC) genome browser [79]. Genomic distribution of CTCF and BORIS ChIP-seq peaks relative to reference genes was performed using the Cis-regulatory Element Annotation System (CEAS) [80]. All ChIP-seq data have been deposited in the Gene Expression Omnibus (GEO) repository with the following accession number [GEO:GSE70764].

### RNA-seq experiments

Two platforms were used for RNA-seq: Illumina for MCF7 and Ion Torrent for K562. For Illumina sequencing, total RNA was extracted from cells using Trizol (Life Technologies) according to the protocol provided by the manufacturer. The RNA quality was assessed using the Agilent Bioanalyzer 2100. The RNA sequencing library preparation and sequencing procedures were carried out according to Illumina protocols with minor modifications. Briefly, poly(A)-mRNA was purified from 10 μg of RNA with streptavidin-coated magnetic beads. After chemical fragmentation, mRNA fragments were reverse-transcribed and converted into double-stranded cDNA. Following end repair and A-tailing, paired-end adaptors were ligated to the ends of the DNA fragments. The ligated products were purified on 2 % agarose gels, and 200–250-bp fragments were selected for downstream enrichment by 18 cycles of PCR followed by purification using a QIAquick PCR purification kit (Qiagen). The enriched libraries were diluted to a final concentration of 5 nM. Each sample was subjected to 50 cycles of sequencing from a single end in Illumina Hiseq2000 Sequencer. For Ion Torrent sequencing, rRNA-depleted RNA was prepared using the RiboMinus Eukaryote System v2 kit (Life Technologies) according to the manufacturer's recommendations. rRNA-depleted RNA (500 ng) was used for library preparation using the Ion Total RNA-seq v2 kit. The enriched libraries were diluted to a final concentration of 11 pM and subjected to sequencing from a single end in a Ion Proton Sequencer.

### Bioinformatic analysis of RNA-seq data

For the Illumina libraries, FASTQ files were mapped to the UCSC Human reference (build hg19) using TopHat2 with the default parameter setting of 20 alignments per read and up to two mismatches per alignment. For Ion Torrent sequencing, FASTQ files were mapped to the UCSC Human reference (build hg19) using two-step alignments. First, the reads were aligned with TopHat2. Second, the unmapped reads from the first step were then extracted and aligned with Bowtie2 with –local mode and the --very-sensitive-local option. In both cases, Illumina and Ion Torrent, the resulting aligned reads (BAM files) were then analyzed with Cufflinks 2.0.0 to estimate transcript relative abundance using the UCSC reference annotated transcripts (build hg19). The expression of each transcript was quantified as the number of reads mapping to a transcript divided by the transcript length in kilobases and the total number of mapped reads in millions (FPKM). RNA-seq data have been deposited in the GEO repository with the following accession number [GEO:GSE70764].

### Published next-generation experiments

ENCODE data for K562 and GM12878 cell lines were used in the study. The list of genomic coordinates for active enhancers (17798) in K562 cells was obtained by overlapping the two sets of ENCODE data [H3K27ac_K562, Bernstein (Broad Institute); p300_K562, Snyder (Stanford)]; overlapping regions that showed the highest enrichment of ChIP-seq tag density of both data sets compared with input were selected. The list of super enhancers (742) was adopted from [41]. The list of active promoters (17,656) was obtained by overlapping of TSSs (RefSeq genes, hg19) extended 2 kb upstream and downstream with the two sets of ENCODE data [H3K4me3_K562, Bernstein (Broad Institute) and RNAPII_K562 Myers (Hudson Alpha)]; the regions positive for both marks were selected.

### Western blotting and immunoprecipitation

Protein extracts were prepared by lysing K562 cells in RIPA Lysis buffer (Millipore) containing 50 mM Tris–HCl, pH 7.4, 1 % Nonidet P-40, 0.25 % sodium deoxycholate, 500 mM NaCl, 1 mM EDTA, 1× protease inhibitor cocktail (Roche Applied Science). For immunoprecipitation analysis, 1.5 mg of total protein was incubated with BORIS monoclonal antibodies, CTCF monoclonal antibodies, or mouse IgG overnight at 4 °C with and without ethidium bromide (100 μg/μl), followed by incubation with 50 μl of Dynabeads M-280 sheep anti-mouse IgG (Life Technologies) for 1 h at room temperature. The immunoprecipitates were collected using a magnetic rack and washed five times with PBS and 0.1 % bovine serum albumin and dissolved in sample buffer for SDS-PAGE. Immunoprecipitated samples were resolved by SDS-PAGE, transferred to a PVDF membrane, and incubated with the indicated antibodies. Detections were performed using ECL reagents.

### Electrophoretic mobility shift assay

DNA fragments encompassing ~200-bp-long sequences derived from either CTCF or BORIS ChIP-seq peaks were synthesized by PCR. The list of primers used in the study is in Additional file 13. In all cases, sequences of PCR fragments were confirmed by sequencing. EMSA was performed as previously described [39]. Briefly, PCR fragments were labeled using ^32^P-γ-ATP with T4 polynucleotide kinase (New England, Biolabs). Protein–DNA complexes were incubated for 1 h at room temperature in binding buffer containing 25 mM Tris pH 7.4, 0.1 mM ZnSO4, 5 mM MgCl_2_, 5 % Nonidet P-40 in PBS, 0.25 mM mercaptoethanol, 10 % glycerol and 0.5 μg of poly dI-dC. Protein–DNA complexes were separated from the unbound probe using 5 % native polyacrylamide gels (PAAG) or 1.2 % agarose gels run in 0.5× Tris-borate-EDTA buffer. Full-length CTCF, full-length BORIS and CTCF 11 ZF domain were translated in vitro using the TnT Quick Coupled Transcription/Translation System (Promega). Nuclear protein extracts were prepared as described in [81]. Pichia CTCF was obtained from AA Vostrov [82].

### In situ proximity ligation assay

Cells seeded on chamber slides (Nunc™ Lab-Tek™ II Chamber Slide™ System), were fixed in 4 % paraformaldehyde for 10 min at 37 °C. Slides were then blocked in 3 % bovine serum albumin (Sigma) in a humidity chamber for 1 h at 37 °C and incubated overnight at 4 °C with mouse and rabbit antibodies: custom monoclonal anti-BORIS and rabbit anti-CTCF (D31H2) (Cell Signaling) in blocking solution. After washing, the slides were incubated with Duolink PLA Rabbit MINUS and PLA Mouse PLUS probes (Olink Bioscience). Ligation and detection were performed using the Duolink reagents kit (Olink Bioscience) according to the manufacturer’s protocol. Fluorescence was detected using a Zeiss Plan Apochromat microscope with a ×63/oil objective. Images were acquired with an Axiocam MRm camera and Imaris software (Bitplane, Co.). The original microscopic images were deposited to Zenodo [83] (10.5281/zenodo.21405).

### Immunofluorescent cell staining

Cells plated onto poly-L lysine coated glass cover slips were fixed with 4 % paraformaldehyde, then washed with PBS. The cells were permeabilized with 0.1 % Triton X-100/PBS for 10 min and subsequently incubated with primary antibodies (anti-CTCF rabbit polyclonal and anti-BORIS mouse monoclonal antibodies; Cell signaling, Inc., Customized). The cells were further probed with fluorescein Texas Red and Alexa Fluor 488 tagged secondary antibodies. DAPI was used for the nuclear counterstain. The fluorescence was recorded using a fluorescence microscope (Zeiss LSM 780). The original microscopic images were deposited to Zenodo [83] (10.5281/zenodo.21405).

### RT-PCR and quantitative PCR

Total RNA was prepared using an RNeasy minikit (Qiagen, Valencia, CA, USA). cDNA was prepared using the SuperScript III first-strand synthesis system (Invitrogen) according to the manufacturer’s protocol. Quantitative PCR was performed using SYBR green PCR master mix (Applied Biosystems, Foster City, CA, USA) and the 7900HT sequence detection system (Applied Biosystems). The primers used in the study are listed in Additional file 13.

## Additional files

Additional file 1: Fig. S1. CTCF and BORIS occupancy in human cancer cell lines. **a** CTCF and BORIS expression in different cell types determined by quantitative PCR. **b** Western blot depicts the relative levels of CTCF and BORIS proteins in nuclear lysates extracted from different cell types. **c** K562 and OVCAR8 cells immunostained with antibodies against CTCF (*green*) and BORIS (*red*). The *merge* panel shows co-localization of CTCF and BORIS proteins in the nucleus. **d** EMSA: either in vitro translated CTCF (*CTCF TNT*) or BORIS (*BORIS TNT*) were incubated with ^32^P-labeled probe. The first lane is a negative control, containing no protein (Free-F). The appearance of a shifted band (*Super Shift*) after adding specific antibodies demonstrated the specificity of DNA–protein interaction and the absence of cross-reactivity between CTCF and BORIS antibodies. **e, f** Venn diagrams depict the numbers and percentages of CTCF (**e**) and BORIS (**f**) bound regions overlapping in K562, OVCAR8 and Delta47 cells. Heatmap shows BORIS (**e**) or CTCF (**f**) occupancy at the invariant CTCF (47,531) or BORIS (17,536) bound regions in three cancer cell types. The tag density was subjected to k-means ranked clustering with two clusters expected. BORIS binds to roughly 30 % of invariant CTSes, while the remaining 70 % of CTSes belong to CTCF-only binding regions. **e** Similar analysis revealed that nearly 70 % of BORIS bound regions showed CTCF occupancy in all three cell lines. **f** Invariant BORIS-only regions mapped in K562 and Delta47 cells were generally occupied by CTCF in OVCAR8 cells (**f**), marked by bracket. **g** Venn diagram depicts the overlapping CTCF bound regions mapped in 38 human cell lines (ENCODE) with BORIS-only bound regions mapped in K562 cells. **h** ChIP-seq tracks show the genomic regions occupied by BORIS alone in K562 cells (BORIS-only) and by both CTCF and BORIS in OVCAR8 and Delta47 cells. (PPTX 1854 kb) Additional file 2: Fig. S2. CTCF and BORIS bind to the same genomic regions simultaneously. **a** The distribution of peak intensity for CTCF (*AllCTCF*) and BORIS (*AllBORIS*) bound regions in the K562 cell line. The peak intensity (tag density) is shown for CTCF peaks that coincide with BORIS peaks (*CTCF&BORIS*), for BORIS peaks that coincide with CTCF peaks (*BORIS&CTCF*), and for CTCF-only and BORIS-only bound regions. Data are shown in logarithmic scale; ****p* < 0.01. **b** Genome browser view of CTCF and BORIS occupancy in K562 and Delta47 cells in combination with ChIP-Re-ChIP-seq data for K562 and Delta47 cells. The *gray frames* highlight CTCF-only, CTCF&BORIS, and BORIS-only bound regions. Only CTCF&BORIS bound regions were detected in ChIP-Re-ChIP data. (PPTX 121 kb) Additional file 3: Fig. S3. EMSA demonstrates the presence of two CTCF binding sites inside BORIS bound regions (*CTCF&BORIS* and *BORIS-only*). **a** *Top panels*: ChIP-seq tracks show CTCF and BORIS occupancies mapped in K562, Delta47 and NHDF cells. From left to right, *TP53* promoter represents CTCF&BORIS bound region; seven CTCF sites residing in the H19-IGF2 imprinting control region are examples of CTCF-only bound regions; the *BMI* promoter represents a BORIS-only bound region; the *PROB1* exon illustrates that a BORIS-only bound region mapped in K562 cells is a CTCF&BORIS bound region in Delta47 cells. *Bottom panels*: binding of in vitro translated 11 ZF domain of CTCF (*11ZF*), full-length CTCF (*CTCF*) and full-length BORIS (*BORIS*) proteins to the *TP53* promoter, one of seven CTCF sites in the H19-IGF2 imprinting control region (fourth CTCF binding site), BMI promoter, and PROB1 exon. In vitro translated luciferase protein was used as a negative control (*Luc*). The DNA complexes with 11 ZF domain, CTCF, and BORIS proteins are indicated by *black*, *red* and *blue arrows*, respectively. The single and double shifts with 11 ZF domain are shown by *single* and *double black arrows*, respectively. The genomic coordinates (hg19) of DNA probes are indicated at the bottom of the gels. **b, c** Five examples of CTCF-only (**b**) and BORIS-only (**c**) bound regions. *Upper panel*: ChIP-seq tracks show CTCF and BORIS occupancies at ten genomic regions mapped in K562, OVCAR8, and Delta47 cells. *Lower panel*: EMSA with the genomic sequences shown on the upper panel. In vitro translated 11ZF domain of CTCF (*11ZF*), full-length CTCF (*CTCF*) and BORIS (*BORIS*) proteins were incubated with the corresponding CTCF-only and BORIS-only bound regions. The single and double shifts with 11 ZF domain are shown by *single* and *double black arrows*, respectively. (PPTX 353 kb) Additional file 4: Fig. S4. CTCF&BORIS bound regions enclose at least two CTCF binding motifs. **a** The motifs identified by MEME for CTCF-only, CTCF&BORIS and BORIS-only bound regions in K562 cells. Additionally, the presence of the CTCF motif was confirmed in 70 % of BORIS-only bound regions. **b, c** The alignment of human and mouse sequences representing five CTCF&BORIS bound regions (**b**) and two CTCF-only bound regions (**c**). The summits of CTCF and BORIS peaks are highlighted by *red* and *blue nucleotides*, respectively. The CTCF motif is shown in *bold italics* and *underlined*. The CTCF motif is indicated with *black* and *blue colors*, depending on sense and antisense orientation, respectively. **d, e** Number of CTCF motifs in the genomic regions, encircling 100 bp upstream and downstream of the summit of CTCF peaks at CTCF&BORIS bound regions (**d**) and from the summit of CTCF-only peaks (**e**). The histogram shows the percentage of CTCF binding regions (y-axis) containing 0 to 7 CTCF motifs (x-axis). **f** The distribution of distances between two CTCF motifs at 1-bp resolution (x-axis) at CTCF&BORIS bound regions (y-axis). The CTCF&BORIS bound regions that have only two CTCF motifs under the peaks were selected for the analysis. (PPTX 3766 kb) Additional file 5: Fig. S5. CTCF&BORIS bound regions enclose at least two CTCF binding sites. **a** Genome browser view of CTCF and BORIS occupancy at seven CTCF&BORIS (*blue bracket*) and seven CTCF-only (*red bracket*) bound regions in K562 and Delta47 cells. *Top panel*: at the top of the genome browser view, the names of sequences are highlighted by *black* or *red colors*, which represent at least two or one CTCF motifs under the peaks, respectively. *Lower panel*: EMSA with the corresponding CTCF&BORIS and CTCF-only binding regions. The ~200-bp ^32^P-labeled probes were incubated with either in vitro translated luciferase (−) or with 11 ZF CTCF domain (*11ZFs*). The slower (shown by *arrow with two red dots*) and faster (*arrow with one red dot*) migrating shifted bands correspond to double and single occupancy, respectively. Of note, the CTCF&BORIS bound regions enclosing one CTCF motif (gene/chromosome name in *red*) are showed double occupancy, similar to CTCF&BORIS bound regions enclosing two CTCF motifs (*black*), while all CTCF-only bound regions showed single occupancy. **b** Three CTCF&BORIS bound regions (TP53, IRF2BP1 and chr17 intergenic regions), each enclosing two CTCF motifs, were split into two fragments and subjected to EMSA. The separation of CTCF motifs resulted in single occupancy of each fragment with one CTCF motif, confirming the presence of two CTCF binding sites in each sequence. **c** 200-bp ^32^P-labeled probes representing *KDM3B*, *FOXA3*, and *TP53* promoters were incubated with nuclear extracts from either K562 or NHDF cells. All lanes contained the indicated nuclear extract, except the first lane for K562, where only free probe is present (F). Nuclear extracts and probe were also incubated with either control mouse IgG (−) or antibodies against CTCF or BORIS. The *red* and *blue arrows* point to the super-shift bands corresponding to CTCF–DNA and BORIS–DNA complexes, respectively. (PPTX 215 kb) Additional file 6: Fig. S6. Comparison of DNaseI digital genomic footprinting (*DGF*) and phastConss46 conservation score at 2xCTSes and 1xCTSes. **a** Heatmaps show DNaseI cleavage density mapped in K562 and NHDF cells at 2xCTSes. 2xCTSes (CTCF&BORIS bound regions in K562 cells) were sorted into 12 different groups (1000 genomic regions in each group) depending on the distance between two CTCF motifs. The distance is shown by *double arrows* and the number of base pairs between two CTCF motifs. The same genomic coordinates were used to plot DNaseI cleavage density mapped in K562 and NHDF cells and for the analysis of average phastCons46 conservation score (shown in the *middle*). The schematic presentation of 2xCTSes with two CTCF motifs (*red boxes*) under one ChIP-seq peak and the proposed occupancy of 2xCTSes by CTCF (*red circle*) and BORIS (*blue circle*) in K562 and NHDF cells are shown at the top of panels. The two CTCF motifs were not sorted based on plus or minus strand. The tag density data were collected within a 400-bp window around the first (*left*) 20-bp CTCF core motifs (0) under a single CTCF ChIP-seq peak. **b** Heatmap shows DNaseI cleavage density mapped in K562 cells at 1xCTSes with CTCF motif on minus (*upper panel*) and plus (*lower panel*) strands. The average phastCons conservation score at the same regions as in DNAseI panels is shown below. **c, d** Heatmaps show DNaseI cleavage density mapped in K562 cells at 2xCTSes. 2xCTSes were separated into two groups with both CTCF motifs located on the plus strand (**c**) or minus strand (**d**). The distance between two CTCF motifs varies from 10 (*bottom*) to 100 bp (*top*). (PPTX 1337 kb) Additional file 7: Fig. S7. Epigenetic profile of two classes of CTCF binding regions in BORIS-positive cells (K562). **a** Heatmaps demonstrate the association of CTCF&BORIS bound regions with active promoters (*H3K4me3* and *RNAPII*, ENCODE) and enhancers (*H3K27ac*, ENCODE) mapped in K562 cells. The tag density was subjected to k-means ranked clustering with five clusters expected. **b** Genome browser view of two examples of Super Enhancers mapped in K562 cells. H3K27ac and p300 tracks were adopted from ENCODE. **c** Scatter plots show the overlapping of CTCF-only and CTCF&BORIS bound regions (*left panel*) and BORIS-only and CTCF&BORIS bound regions (*right panel*) with ChIP-seq data available for K562 cells by ENCODE. The *dots* represent the percentage of CTCF-only, BORIS-only (x-axis), and CTCF&BORIS (y-axis) bound regions overlapping with each factor mapped by ChIP-seq in K562 cells. The *red dots* are labeled with the name of the factor mapped by ChIP-seq. **d** The percentage of BORIS-only, CTCF&BORIS and CTCF-only bound regions (y-axis) overlapping with each factor (x-axis). **e** Heatmap demonstrates the enrichment of RNAPII, CAGEs, H3K4me3, H2AZ, H3K27ac, ZNF143, and SMC3 ChIP-seq data (ENCODE data) at CTCF&BORIS bound regions in contrast to CTCF-only bound regions. The tag density of ChIP-seq data was collected within a 10-kb window around the summit of CTCF peaks mapped in K562 cells. The collected data were subjected to k-means clustering using linear normalization. **f** Average tag density (tags/10 million) of multiple factors mapped by ChIP-seq in K562 cells (ENCODE data) across BORIS-only (*blue*), CTCF-only (*red*) and CTCF&BORIS (*purple*) bound regions mapped in K562 cells. The names of factors used in ChIP-seq are labeled on the top of the each plot. **g** Histogram shows the genomic distribution of CTCF and BORIS occupancy at promoters plus the 5’ UTR (3 kb up- and downstream of TSSs), gene bodies, and intergenic regions. (PPTX 680 kb) Additional file 8: Fig. S8. Epigenetic profile of two classes of CTCF binding regions in BORIS-negative cells (GM12878). **a** Average tag density (tags/10 million) of CTCF, RNAPII, DNaseI digestion, H3K4me3, H3K27ac, and RAD21 (mapped by ChIP-seq in GM12878 cells, ENCODE data) at 2xCTSes (*blue*) and 1xCTSes (*red*). The data were normalized to the number of mapped reads and binding regions. **b** *Upper panel*: scatter plot shows overlapping of 2xCTSes (y-axis) and 1xCTSes (x-axis) with multiple ENCODE data available for GM12878. Similar to CTCF&BORIS and CTCF-only bound regions in K562 (Fig. S7c, left panel in Additional file 7), no correlation (R^2^ = 0.41) was found between 2xCTSes and 1xCTSes with respect to co-occupancy with the majority of transcription factors, histone modifications, and chromatin remodeling factors. *Middle panel*: scatter plot shows an overlapping of 1xCTSes mapped in K562 (y-axis) and in GM12878 cells (x-axis) with ENCODE data for K562 and GM12878 cells, respectively. 1xCTSes of GM12878 and K562 cells were correlated with each other (R^2^ = 0.98) with respect to co-occupancy with the majority of ENCODE data common for the two cell lines. *Lower panel*: scatter plot shows the overlapping of 2xCTSes in K562 (y-axis) and in GM12878 (x-axis) with ENCODE data for K562 and GM12878 cells (R^2^ = 0.93), respectively. (PPTX 220 kb) Additional file 9: Fig. S9. BORIS occupancy in cancer cells recapitulates the BORIS occupancy in germ cells. **a** Average tag density of CTCF (*red*) and BORIS (*blue*) occupancies mapped in OVCAR8 and Delta47 cells across the conserved mouse binding regions (*upper panel*). **b** Gene tracks representing the examples of conserved BORIS-only, CTCF-only and CTCF&BORIS bound regions in germline (round spermatids) and cancer cells (K562). (PPTX 104 kb) Additional file 10: Fig. S10. BORIS is involved in transcriptional program of cancers. **a** The sequence recognized by ZFN and cleaved by Fok I is shown by *black* and *red letters*, respectively. **b** Surveyor assay (*CEL-I*) for ZFN-induced mutations in the BORIS gene. The proportions of wild-type and mutant alleles are shown as a table at the bottom of the gel. **c** Surveyor assay shows ten single-cell clones with the mutated BORIS locus. **d** Western blot shows the level of BORIS protein in wild type (*wt*) and ten mutant clones from panel (c). **e** K562 cells transfected with ZFN produced at least ten times less colonies in soft agar compared with control. **f** Western blot shows the level of BORIS protein in K562 before (0) and after phorbol 12*-*myristate 13*-*acetate (PMA) treatment. Wright–Giemsa staining analysis of K562 transfected with either control vector or ZFN in comparison with K562 treated with PMA for 3 days. Both transfection of K562 with ZFN and treatment with PMA showed morphologic changes related to megakaryocytic differentiation. **h** *Upper panel*: ChIP-seq and RNA-seq tracks show CTCF and BORIS occupancy at the *WISP2* gene and dowregulation of *WISP2* expression upon BORIS induction in MCF7 cells. *Lower panel*: RNA-seq RPKM values for the *WISP2* gene expression. **i** Downregulation of inflammatory response pathway in response to stable BORIS expression in MCF7 cells. **k** ChIP-seq tracks show CTCF and BORIS occupancy at *GAL3ST1*, *FOXA3*, and *PRAME* loci in NHDF, OVCAR8, Delta47, and K562 cells. The tracks are labeled with the molecules against which antibodies were directed and cell lines used in ChIP-seq. RNA-Seq tracks demonstrate gene expression in BORIS-positive cells (K562) in contrast to BORIS-negative cells (NHEK and NHDF). **l** Quantitative PCR analysis of *GAL3ST1*, *FOXA3*, and *PRAME* gene expression in BORIS-positive cells (Testes, K562, OVCAR8, Delta47) and BORIS-negative cells (GM12878, NHDF, NHEK). (PPTX 1328 kb) Additional file 11: Table S1. Pathways altered with BORIS downregulation in K562 cells. **Table S2** Functional annotation of genes that changed transcription upon BORIS downregulation in K562 cells. **Table S3** The top diseases and functions enriched for the genes that changed transcription upon BORIS downregulation in K562 cells. **Table S4** Pathways altered with BORIS induction in MCF7 cells. **Table S5** Functional annotation of genes that changed transcription upon BORIS induction in MCF7 cells (*Clone1*). **Table S6** The top diseases and functions enriched for the genes that changed transcription upon BORIS induction in MCF7 cells (*Clone1*). **Table S7** Comparison of functional annotation of genes that changed transcription upon BORIS depletion (K562) and BORIS induction (MCF7, *Clone1*). **Table S8** Comparison of canonical pathways that altered upon BORIS depletion (K562) and BORIS induction (MCF7, *Clone1*). (XLSX 257 kb) Additional file 12: Fig. S11. CTCF and BORIS bound regions enriched at the anchors of cell-type specific transcriptional loops in K562 cells compared with MCF7 cells. **a** Heatmap shows the enrichment of BORIS, CTCF, and RNAPII occupancy in K562 cells at the left and right anchors of K562-specific loops. **b** Heatmap shows the enrichment of CTCF and RNAPII occupancy in MCF7 cells at the anchors of K562-specific loops, but the interactions mediated by CTCF were different in MCF7 cells compared with K562 cells. **c** Genomic view of K562-specific long-range chromatin interactions at the COMMD7-DNMT3B-MAPRE1 locus. The tracks are labeled with the molecules against which antibodies were directed in ChIP-seq in both K562 and MCF7 cells. ChIA-PET data are presented as *red lines with bold anchors* and summarized by brackets on the top of the gene tracks. **d** Model of cluster and single CTCF binding classes and their modulation by BORIS in BORIS-positive cells. The model is based on ChIP-seq and ChIA-PET data for K562 and MCF7 cells at the *DNMT3B* locus (**c**), but also observed in *TP53*, *FOXA3*, *PRAME*, *KDM3B*, *MDM2*, *BBC3* and other loci. (PPTX 209 kb) Additional file 13: Table S9 The primers used in the study for amplification of EMSA probes and for quantitative PCR. (DOCX 17 kb)

## Abbreviations

BORIS: Brother of the regulator of imprinted sites
bp: base pair
ChIP: chromatin immunoprecipitation
CTCF: CCCTC-binding factor
CTS: CTCF target site
DAPI: 4′,6-diamidino-2-phenylindole
EMSA: electrophoretic mobility shift assay
GEO: Gene Expression Omnibus
ISPLA: in situ proximity ligation assay
MACS: Model-based Analysis for ChIP-seq
MIA: methylation interference assay
NHDF: normal human dermal fibroblast
PBS: phosphate buffered saline
PCR: polymerase chain reaction
RNAPII: RNA polymerase II
TSS: transcription start site
UCSC: University of California, Santa Cruz
UTR: untranslated region
ZF: zinc finger
ZFN: zinc finger nuclease
