# Supplementary material for: Comparative analyses of CTCF and BORIS occupancies uncover two distinct classes of CTCF binding genomic regions
Source: Genome Biol. 2015 Aug 14;16(1):161. doi: 10.1186/s13059-015-0736-8 (PMC4562119; doi:10.1186/s13059-015-0736-8)
Supplement: Additional file 1: Fig. S1. — CTCF and BORIS occupancy in human cancer cell lines. a CTCF and BORIS expression in different cell types determined by quantitative PCR. b Western blot depicts the relative levels of CTCF and BORIS proteins in nuclear lysates extracted from different cell types. c K562 and OVCAR8 cells immunostained with antibodies against CTCF (green) and BORIS (red). The merge panel shows co-localization of CTCF and BORIS proteins in the nucleus. d EMSA: either in vitro translated CTCF (CTCF TNT) or BORIS (BORIS TNT) were incubated with 32P-labeled probe. The first lane is a negative control, containing no protein (Free-F). The appearance of a shifted band (Super Shift) after adding specific antibodies demonstrated the specificity of DNA–protein interaction and the absence of cross-reactivity between CTCF and BORIS antibodies. e, f Venn diagrams depict the numbers and percentages of CTCF (e) and BORIS (f) bound regions overlapping in K562, OVCAR8 and Delta47 cells. Heatmap shows BORIS (e) or CTCF (f) occupancy at the invariant CTCF (47,531) or BORIS (17,536) bound regions in three cancer cell types. The tag density was subjected to k-means ranked clustering with two clusters expected. BORIS binds to roughly 30 % of invariant CTSes, while the remaining 70 % of CTSes belong to CTCF-only binding regions. e Similar analysis revealed that nearly 70 % of BORIS bound regions showed CTCF occupancy in all three cell lines. f Invariant BORIS-only regions mapped in K562 and Delta47 cells were generally occupied by CTCF in OVCAR8 cells (f), marked by bracket. g Venn diagram depicts the overlapping CTCF bound regions mapped in 38 human cell lines (ENCODE) with BORIS-only bound regions mapped in K562 cells. h ChIP-seq tracks show the genomic regions occupied by BORIS alone in K562 cells (BORIS-only) and by both CTCF and BORIS in OVCAR8 and Delta47 cells. (PPTX 1854 kb) [file 13059_2015_736_MOESM1_ESM.pptx]

## Slide 1
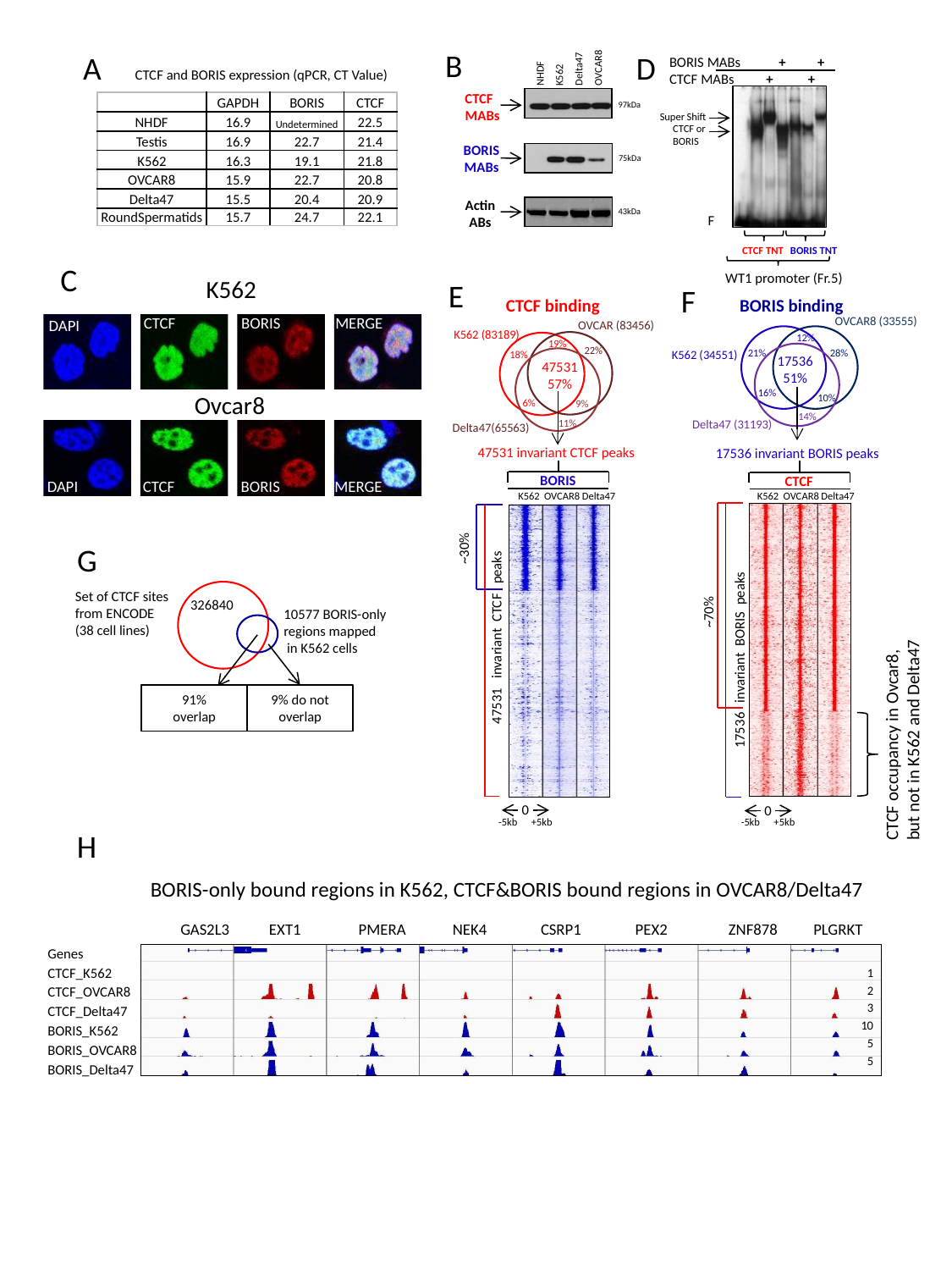

NHDF
K562
Delta47
OVCAR8
B
 BORIS MABs + +
 CTCF MABs + +
CTCF
MABs
97kDa
Super Shift
CTCF or
BORIS
BORIS
MABs
75kDa
Actin
ABs
43kDa
F
 CTCF TNT BORIS TNT
WT1 promoter (Fr.5)
A
D
CTCF and BORIS expression (qPCR, CT Value)
| | GAPDH | BORIS | CTCF |
| --- | --- | --- | --- |
| NHDF | 16.9 | Undetermined | 22.5 |
| Testis | 16.9 | 22.7 | 21.4 |
| K562 | 16.3 | 19.1 | 21.8 |
| OVCAR8 | 15.9 | 22.7 | 20.8 |
| Delta47 | 15.5 | 20.4 | 20.9 |
| RoundSpermatids | 15.7 | 24.7 | 22.1 |
C
K562
E
CTCF binding
OVCAR (83456)
K562 (83189)
19%
22%
18%
47531
57%
6%
9%
11%
Delta47(65563)
47531 invariant CTCF peaks
BORIS
K562 OVCAR8 Delta47
~30%
47531 invariant CTCF peaks
0
-5kb +5kb
F
BORIS binding
OVCAR8 (33555)
28%
21%
K562 (34551)
17536
51%
16%
10%
14%
Delta47 (31193)
CTCF
BORIS
MERGE
DAPI
12%
Ovcar8
17536 invariant BORIS peaks
CTCF
DAPI
CTCF
BORIS
MERGE
 K562 OVCAR8 Delta47
G
Set of CTCF sites from ENCODE
(38 cell lines)
326840
10577 BORIS-only regions mapped
 in K562 cells
91%
overlap
9% do not overlap
~70%
17536 invariant BORIS peaks
CTCF occupancy in Ovcar8,
but not in K562 and Delta47
0
-5kb +5kb
H
BORIS-only bound regions in K562, CTCF&BORIS bound regions in OVCAR8/Delta47
GAS2L3 EXT1 PMERA NEK4 CSRP1 PEX2 ZNF878 PLGRKT
Genes
CTCF_K562
CTCF_OVCAR8
CTCF_Delta47
BORIS_K562
BORIS_OVCAR8
BORIS_Delta47
 1
 2
 3
10
 5
 5
