# Supplementary material for: Comparative analyses of CTCF and BORIS occupancies uncover two distinct classes of CTCF binding genomic regions
Source: Genome Biol. 2015 Aug 14;16(1):161. doi: 10.1186/s13059-015-0736-8 (PMC4562119; doi:10.1186/s13059-015-0736-8)
Supplement: Additional file 2: Fig. S2. — CTCF and BORIS bind to the same genomic regions simultaneously. a The distribution of peak intensity for CTCF (AllCTCF) and BORIS (AllBORIS) bound regions in the K562 cell line. The peak intensity (tag density) is shown for CTCF peaks that coincide with BORIS peaks (CTCF&BORIS), for BORIS peaks that coincide with CTCF peaks (BORIS&CTCF), and for CTCF-only and BORIS-only bound regions. Data are shown in logarithmic scale; ***p < 0.01. b Genome browser view of CTCF and BORIS occupancy in K562 and Delta47 cells in combination with ChIP-Re-ChIP-seq data for K562 and Delta47 cells. The gray frames highlight CTCF-only, CTCF&BORIS, and BORIS-only bound regions. Only CTCF&BORIS bound regions were detected in ChIP-Re-ChIP data. (PPTX 121 kb) [file 13059_2015_736_MOESM2_ESM.pptx]

## Slide 1
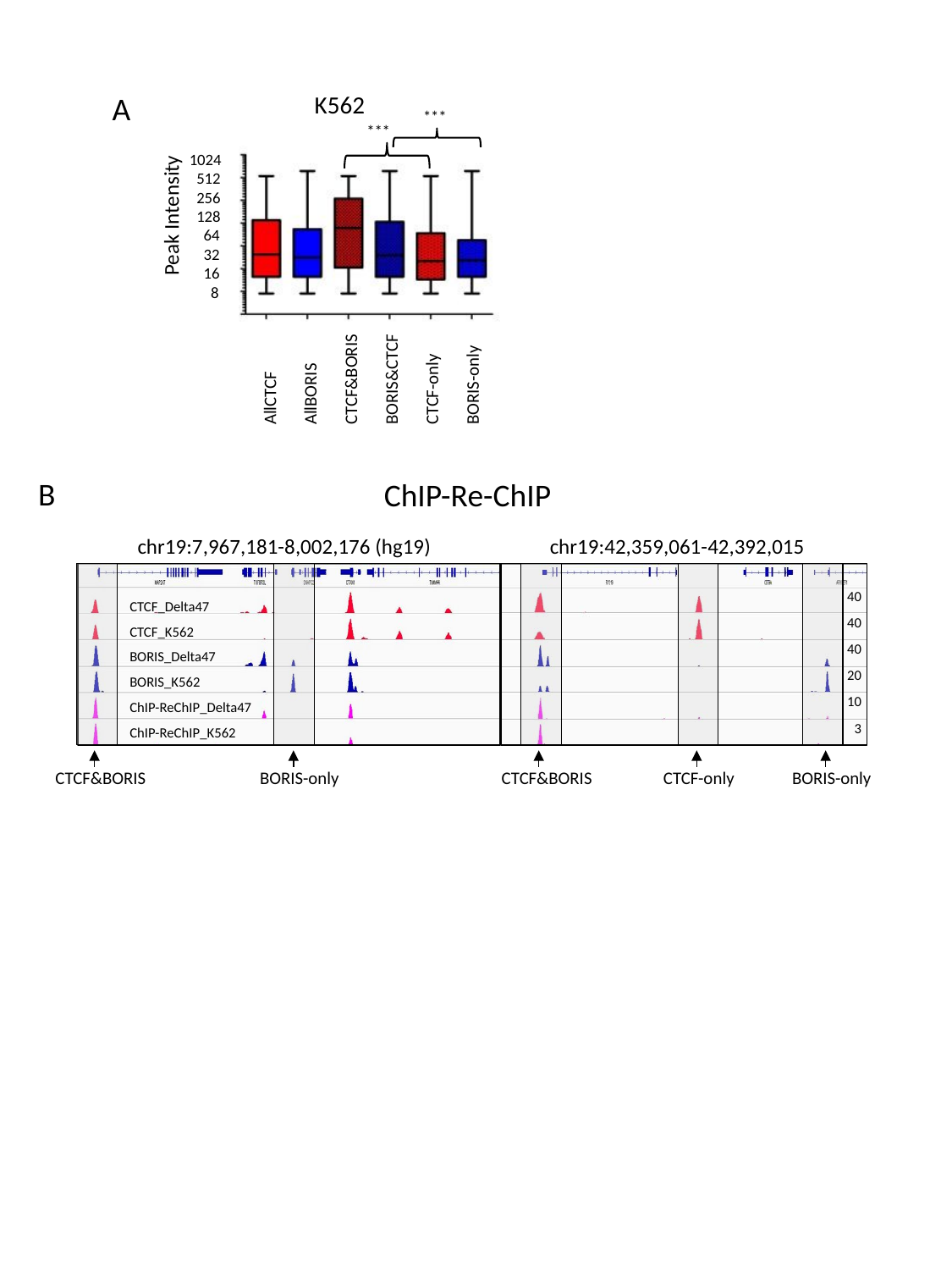

K562
1024
 512
 256
 128
 64
 32
 16
 8
Peak Intensity
AllCTCF
AllBORIS
CTCF&BORIS
BORIS&CTCF
CTCF-only
BORIS-only
***
***
A
B
ChIP-Re-ChIP
chr19:7,967,181-8,002,176 (hg19)
chr19:42,359,061-42,392,015
40
40
40
20
10
3
CTCF_Delta47
CTCF_K562
BORIS_Delta47
BORIS_K562
ChIP-ReChIP_Delta47
ChIP-ReChIP_K562
CTCF&BORIS
BORIS-only
CTCF&BORIS
CTCF-only
BORIS-only
Figure S16
