# Supplementary material for: Comparative analyses of CTCF and BORIS occupancies uncover two distinct classes of CTCF binding genomic regions
Source: Genome Biol. 2015 Aug 14;16(1):161. doi: 10.1186/s13059-015-0736-8 (PMC4562119; doi:10.1186/s13059-015-0736-8)
Supplement: Additional file 3: Fig. S3. — EMSA demonstrates the presence of two CTCF binding sites inside BORIS bound regions (CTCF&BORIS and BORIS-only). a Top panels: ChIP-seq tracks show CTCF and BORIS occupancies mapped in K562, Delta47 and NHDF cells. From left to right, TP53 promoter represents CTCF&BORIS bound region; seven CTCF sites residing in the H19-IGF2 imprinting control region are examples of CTCF-only bound regions; the BMI promoter represents a BORIS-only bound region; the PROB1 exon illustrates that a BORIS-only bound region mapped in K562 cells is a CTCF&BORIS bound region in Delta47 cells. Bottom panels: binding of in vitro translated 11 ZF domain of CTCF (11ZF), full-length CTCF (CTCF) and full-length BORIS (BORIS) proteins to the TP53 promoter, one of seven CTCF sites in the H19-IGF2 imprinting control region (fourth CTCF binding site), BMI promoter, and PROB1 exon. In vitro translated luciferase protein was used as a negative control (Luc). The DNA complexes with 11 ZF domain, CTCF, and BORIS proteins are indicated by black, red and blue arrows, respectively. The single and double shifts with 11 ZF domain are shown by single and double black arrows, respectively. The genomic coordinates (hg19) of DNA probes are indicated at the bottom of the gels. b, c Five examples of CTCF-only (b) and BORIS-only (c) bound regions. Upper panel: ChIP-seq tracks show CTCF and BORIS occupancies at ten genomic regions mapped in K562, OVCAR8, and Delta47 cells. Lower panel: EMSA with the genomic sequences shown on the upper panel. In vitro translated 11ZF domain of CTCF (11ZF), full-length CTCF (CTCF) and BORIS (BORIS) proteins were incubated with the corresponding CTCF-only and BORIS-only bound regions. The single and double shifts with 11 ZF domain are shown by single and double black arrows, respectively. (PPTX 353 kb) [file 13059_2015_736_MOESM3_ESM.pptx]

## Slide 1
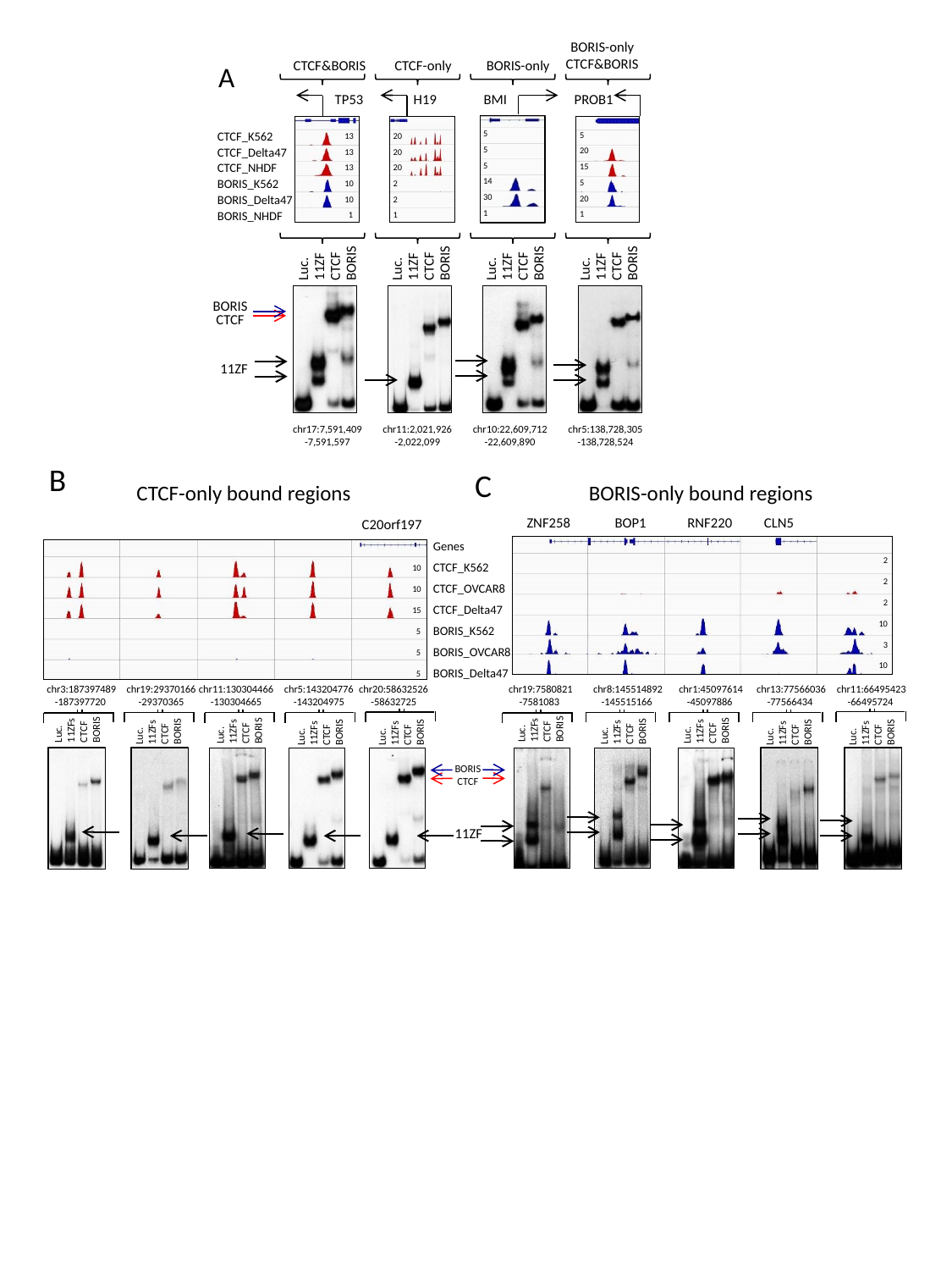

BORIS-only
CTCF&BORIS
A
CTCF&BORIS CTCF-only BORIS-only
TP53
 H19
BMI
PROB1
5
5
5
14
30
1
5
20
15
5
20
1
20
20
20
2
2
1
CTCF_K562
CTCF_Delta47
CTCF_NHDF
BORIS_K562
BORIS_Delta47
BORIS_NHDF
Luc.
11ZF
CTCF
BORIS
Luc.
11ZF
CTCF
BORIS
Luc.
11ZF
CTCF
BORIS
Luc.
11ZF
CTCF
BORIS
BORIS
CTCF
11ZF
chr17:7,591,409
-7,591,597
chr11:2,021,926
-2,022,099
chr10:22,609,712
-22,609,890
chr5:138,728,305
-138,728,524
13
13
13
10
10
 1
B
C
CTCF-only bound regions
BORIS-only bound regions
ZNF258 BOP1 RNF220 CLN5
C20orf197
Genes
CTCF_K562
CTCF_OVCAR8
CTCF_Delta47
BORIS_K562
BORIS_OVCAR8
BORIS_Delta47
 2
 2
 2
10
 3
 10
 10
 10
 15
 5
 5
 5
chr3:187397489
-187397720
chr19:29370166
-29370365
chr11:130304466
-130304665
chr5:143204776
-143204975
chr20:58632526
-58632725
chr19:7580821
-7581083
chr8:145514892
-145515166
chr1:45097614
-45097886
chr13:77566036
-77566434
chr11:66495423
-66495724
Luc.
11ZFs
CTCF
BORIS
Luc.
11ZFs
CTCF
BORIS
Luc.
11ZFs
CTCF
BORIS
Luc.
11ZFs
CTCF
BORIS
Luc.
11ZFs
CTCF
BORIS
Luc.
11ZFs
CTCF
BORIS
Luc.
11ZFs
CTCF
BORIS
Luc.
11ZFs
CTCF
BORIS
Luc.
11ZFs
CTCF
BORIS
Luc.
11ZFs
CTCF
BORIS
BORIS
CTCF
11ZF
Figure S16
