# Supplementary material for: Comparative analyses of CTCF and BORIS occupancies uncover two distinct classes of CTCF binding genomic regions
Source: Genome Biol. 2015 Aug 14;16(1):161. doi: 10.1186/s13059-015-0736-8 (PMC4562119; doi:10.1186/s13059-015-0736-8)
Supplement: Additional file 4: Fig. S4. — CTCF&BORIS bound regions enclose at least two CTCF binding motifs. a The motifs identified by MEME for CTCF-only, CTCF&BORIS and BORIS-only bound regions in K562 cells. Additionally, the presence of the CTCF motif was confirmed in 70 % of BORIS-only bound regions. b, c The alignment of human and mouse sequences representing five CTCF&BORIS bound regions (b) and two CTCF-only bound regions (c). The summits of CTCF and BORIS peaks are highlighted by red and blue nucleotides, respectively. The CTCF motif is shown in bold italics and underlined. The CTCF motif is indicated with black and blue colors, depending on sense and antisense orientation, respectively. d, e Number of CTCF motifs in the genomic regions, encircling 100 bp upstream and downstream of the summit of CTCF peaks at CTCF&BORIS bound regions (d) and from the summit of CTCF-only peaks (e). The histogram shows the percentage of CTCF binding regions (y-axis) containing 0 to 7 CTCF motifs (x-axis). f The distribution of distances between two CTCF motifs at 1-bp resolution (x-axis) at CTCF&BORIS bound regions (y-axis). The CTCF&BORIS bound regions that have only two CTCF motifs under the peaks were selected for the analysis. (PPTX 3766 kb) [file 13059_2015_736_MOESM4_ESM.pptx]

## Slide 1
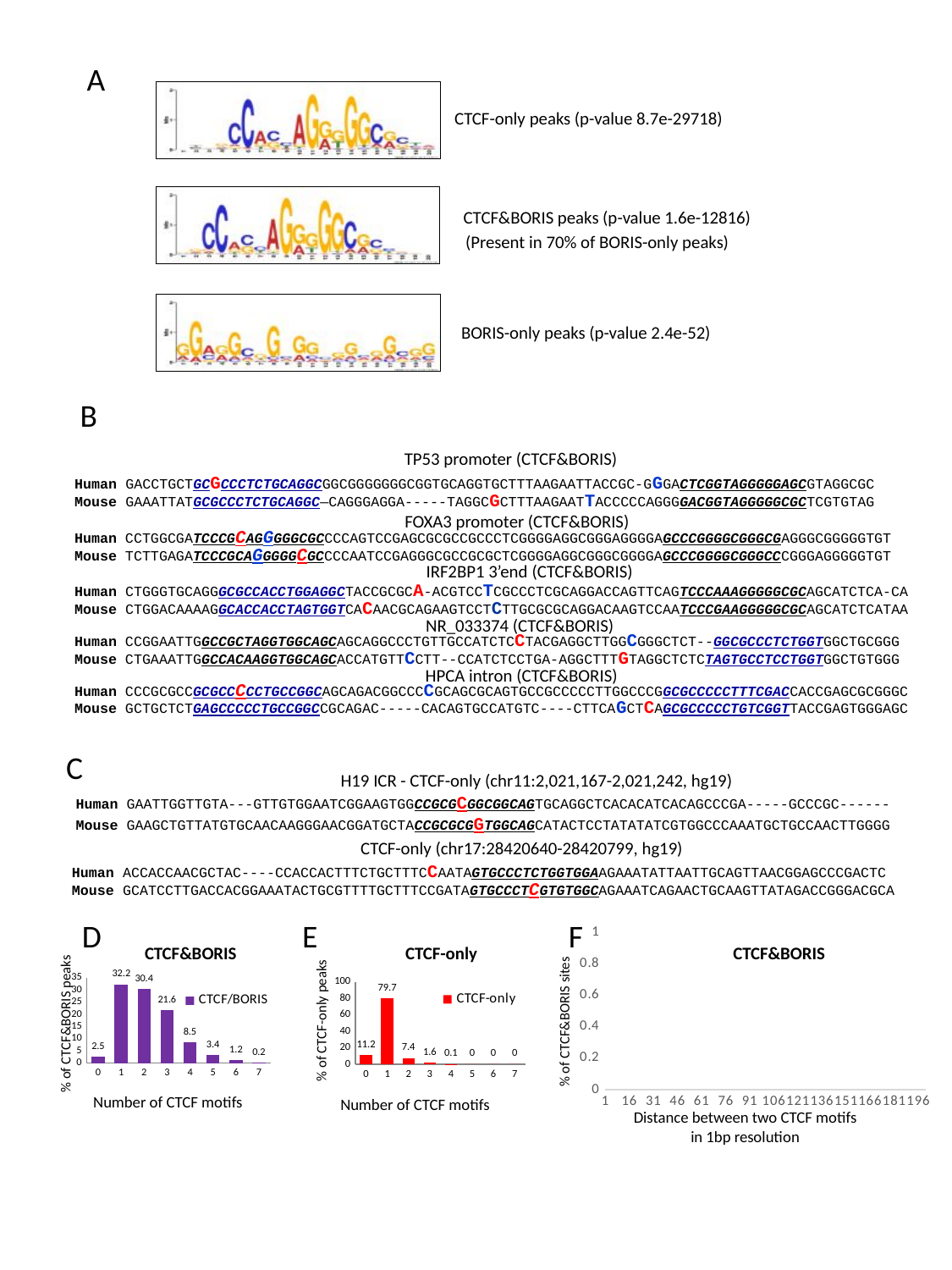

A
CTCF-only peaks (p-value 8.7e-29718)
CTCF&BORIS peaks (p-value 1.6e-12816)
(Present in 70% of BORIS-only peaks)
 BORIS-only peaks (p-value 2.4e-52)
B
TP53 promoter (CTCF&BORIS)
Human GACCTGCTGCGCCCTCTGCAGGCGGCGGGGGGGCGGTGCAGGTGCTTTAAGAATTACCGC-GGGACTCGGTAGGGGGAGCGTAGGCGC
Mouse GAAATTATGCGCCCTCTGCAGGC—CAGGGAGGA-----TAGGCGCTTTAAGAATTACCCCCAGGGGACGGTAGGGGGCGCTCGTGTAG
FOXA3 promoter (CTCF&BORIS)
Human CCTGGCGATCCCGCAGGGGGCGCCCCAGTCCGAGCGCGCCGCCCTCGGGGAGGCGGGAGGGGAGCCCGGGGCGGGCGAGGGCGGGGGTGT
Mouse TCTTGAGATCCCGCAGGGGGCGCCCCAATCCGAGGGCGCCGCGCTCGGGGAGGCGGGCGGGGAGCCCGGGGCGGGCCCGGGAGGGGGTGT
IRF2BP1 3’end (CTCF&BORIS)
Human CTGGGTGCAGGGCGCCACCTGGAGGCTACCGCGCA-ACGTCCTCGCCCTCGCAGGACCAGTTCAGTCCCAAAGGGGGCGCAGCATCTCA-CA
Mouse CTGGACAAAAGGCACCACCTAGTGGTCACAACGCAGAAGTCCTCTTGCGCGCAGGACAAGTCCAATCCCGAAGGGGGCGCAGCATCTCATAA
NR_033374 (CTCF&BORIS)
Human CCGGAATTGGCCGCTAGGTGGCAGCAGCAGGCCCTGTTGCCATCTCCTACGAGGCTTGGCGGGCTCT--GGCGCCCTCTGGTGGCTGCGGG
Mouse CTGAAATTGGCCACAAGGTGGCAGCACCATGTTCCTT--CCATCTCCTGA-AGGCTTTGTAGGCTCTCTAGTGCCTCCTGGTGGCTGTGGG
HPCA intron (CTCF&BORIS)
Human CCCGCGCCGCGCCCCCTGCCGGCAGCAGACGGCCCCGCAGCGCAGTGCCGCCCCCTTGGCCCGGCGCCCCCTTTCGACCACCGAGCGCGGGC
Mouse GCTGCTCTGAGCCCCCTGCCGGCCGCAGAC-----CACAGTGCCATGTC----CTTCAGCTCAGCGCCCCCTGTCGGTTACCGAGTGGGAGC
C
H19 ICR - CTCF-only (chr11:2,021,167-2,021,242, hg19)
Human GAATTGGTTGTA---GTTGTGGAATCGGAAGTGGCCGCGCGGCGGCAGTGCAGGCTCACACATCACAGCCCGA-----GCCCGC------
Mouse GAAGCTGTTATGTGCAACAAGGGAACGGATGCTACCGCGCGGTGGCAGCATACTCCTATATATCGTGGCCCAAATGCTGCCAACTTGGGG
CTCF-only (chr17:28420640-28420799, hg19)
Human ACCACCAACGCTAC----CCACCACTTTCTGCTTTCCAATAGTGCCCTCTGGTGGAAGAAATATTAATTGCAGTTAACGGAGCCCGACTC
Mouse GCATCCTTGACCACGGAAATACTGCGTTTTGCTTTCCGATAGTGCCCTCGTGTGGCAGAAATCAGAACTGCAAGTTATAGACCGGGACGCA
D E F
### Chart
| Category | |
|---|---|CTCF&BORIS
CTCF-only
CTCF&BORIS
### Chart
| Category | |
|---|---|
| 0 | 11.2 |
| 1 | 79.7 |
| 2 | 7.4 |
| 3 | 1.6 |
| 4 | 0.1 |
| 5 | 0.0 |
| 6 | 0.0 |
| 7 | 0.0 |
### Chart
| Category | |
|---|---|
| 0 | 2.5 |
| 1 | 32.2 |
| 2 | 30.4 |
| 3 | 21.6 |
| 4 | 8.5 |
| 5 | 3.4 |
| 6 | 1.2 |
| 7 | 0.2 |% of CTCF-only peaks
% of CTCF&BORIS sites
% of CTCF&BORIS peaks
Number of CTCF motifs
Number of CTCF motifs
Distance between two CTCF motifs
in 1bp resolution
