# Supplementary material for: Comparative analyses of CTCF and BORIS occupancies uncover two distinct classes of CTCF binding genomic regions
Source: Genome Biol. 2015 Aug 14;16(1):161. doi: 10.1186/s13059-015-0736-8 (PMC4562119; doi:10.1186/s13059-015-0736-8)
Supplement: Additional file 5: Fig. S5. — CTCF&BORIS bound regions enclose at least two CTCF binding sites. a Genome browser view of CTCF and BORIS occupancy at seven CTCF&BORIS (blue bracket) and seven CTCF-only (red bracket) bound regions in K562 and Delta47 cells. Top panel: at the top of the genome browser view, the names of sequences are highlighted by black or red colors, which represent at least two or one CTCF motifs under the peaks, respectively. Lower panel: EMSA with the corresponding CTCF&BORIS and CTCF-only binding regions. The ~200-bp 32P-labeled probes were incubated with either in vitro translated luciferase (−) or with 11 ZF CTCF domain (11ZFs). The slower (shown by arrow with two red dots) and faster (arrow with one red dot) migrating shifted bands correspond to double and single occupancy, respectively. Of note, the CTCF&BORIS bound regions enclosing one CTCF motif (gene/chromosome name in red) are showed double occupancy, similar to CTCF&BORIS bound regions enclosing two CTCF motifs (black), while all CTCF-only bound regions showed single occupancy. b Three CTCF&BORIS bound regions (TP53, IRF2BP1 and chr17 intergenic regions), each enclosing two CTCF motifs, were split into two fragments and subjected to EMSA. The separation of CTCF motifs resulted in single occupancy of each fragment with one CTCF motif, confirming the presence of two CTCF binding sites in each sequence. c 200-bp 32P-labeled probes representing KDM3B, FOXA3, and TP53 promoters were incubated with nuclear extracts from either K562 or NHDF cells. All lanes contained the indicated nuclear extract, except the first lane for K562, where only free probe is present (F). Nuclear extracts and probe were also incubated with either control mouse IgG (−) or antibodies against CTCF or BORIS. The red and blue arrows point to the super-shift bands corresponding to CTCF–DNA and BORIS–DNA complexes, respectively. (PPTX 215 kb) [file 13059_2015_736_MOESM5_ESM.pptx]

## Slide 1
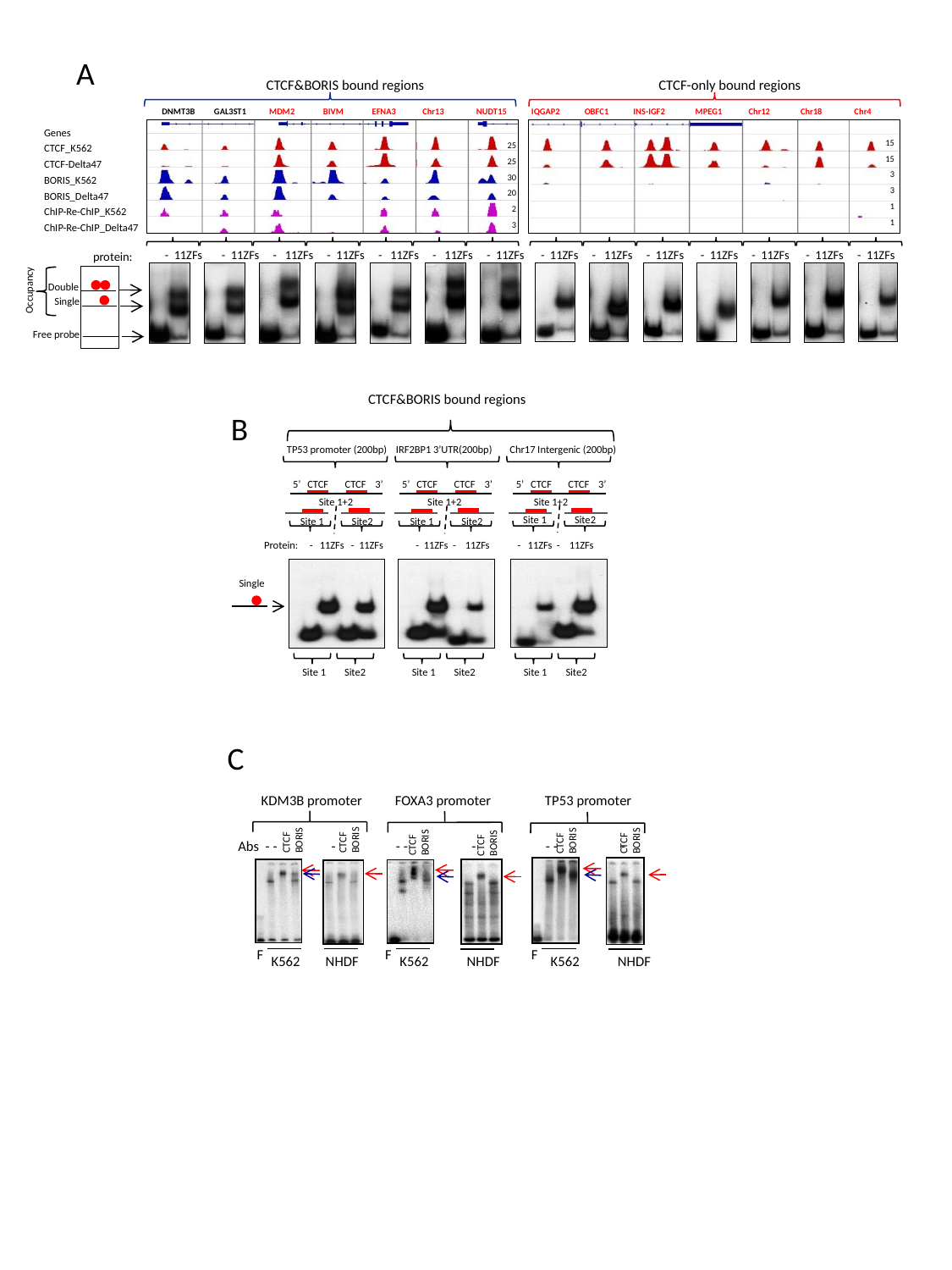

A
CTCF&BORIS bound regions CTCF-only bound regions
DNMT3B GAL3ST1 MDM2 BIVM EFNA3 Chr13 NUDT15 IQGAP2 OBFC1 INS-IGF2 MPEG1 Chr12 Chr18 Chr4
Genes
CTCF_K562
CTCF-Delta47
BORIS_K562
BORIS_Delta47
ChIP-Re-ChIP_K562
ChIP-Re-ChIP_Delta47
15
15
3
3
1
1
25
25
30
20
2
3
 - 11ZFs - 11ZFs - 11ZFs - 11ZFs - 11ZFs - 11ZFs - 11ZFs - 11ZFs - 11ZFs - 11ZFs - 11ZFs - 11ZFs - 11ZFs - 11ZFs
protein:
Double
Occupancy
Single
Free probe
CTCF&BORIS bound regions
B
TP53 promoter (200bp)
CTCF CTCF
5’ 3’
IRF2BP1 3’UTR(200bp)
CTCF CTCF
5’ 3’
Chr17 Intergenic (200bp)
CTCF CTCF
5’ 3’
Protein: - 11ZFs - 11ZFs - 11ZFs - 11ZFs - 11ZFs - 11ZFs
Site 1+2 Site 1+2 Site 1+2
Site 1 Site2
Site 1 Site2
Site 1 Site2
Single
Site 1 Site2
Site 1 Site2
Site 1 Site2
C
KDM3B promoter
FOXA3 promoter
TP53 promoter
CTCF
BORIS
CTCF
BORIS
CTCF
BORIS
CTCF
BORIS
CTCF
BORIS
CTCF
BORIS
F
F
F
Abs - - - - - - - - -
 K562 NHDF K562 NHDF K562 NHDF
