# Supplementary figures and images for: Comparative analyses of CTCF and BORIS occupancies uncover two distinct classes of CTCF binding genomic regions
Source: Genome Biol. 2015 Aug 14;16(1):161. doi: 10.1186/s13059-015-0736-8 (PMC4562119; doi:10.1186/s13059-015-0736-8)

## Slide 1
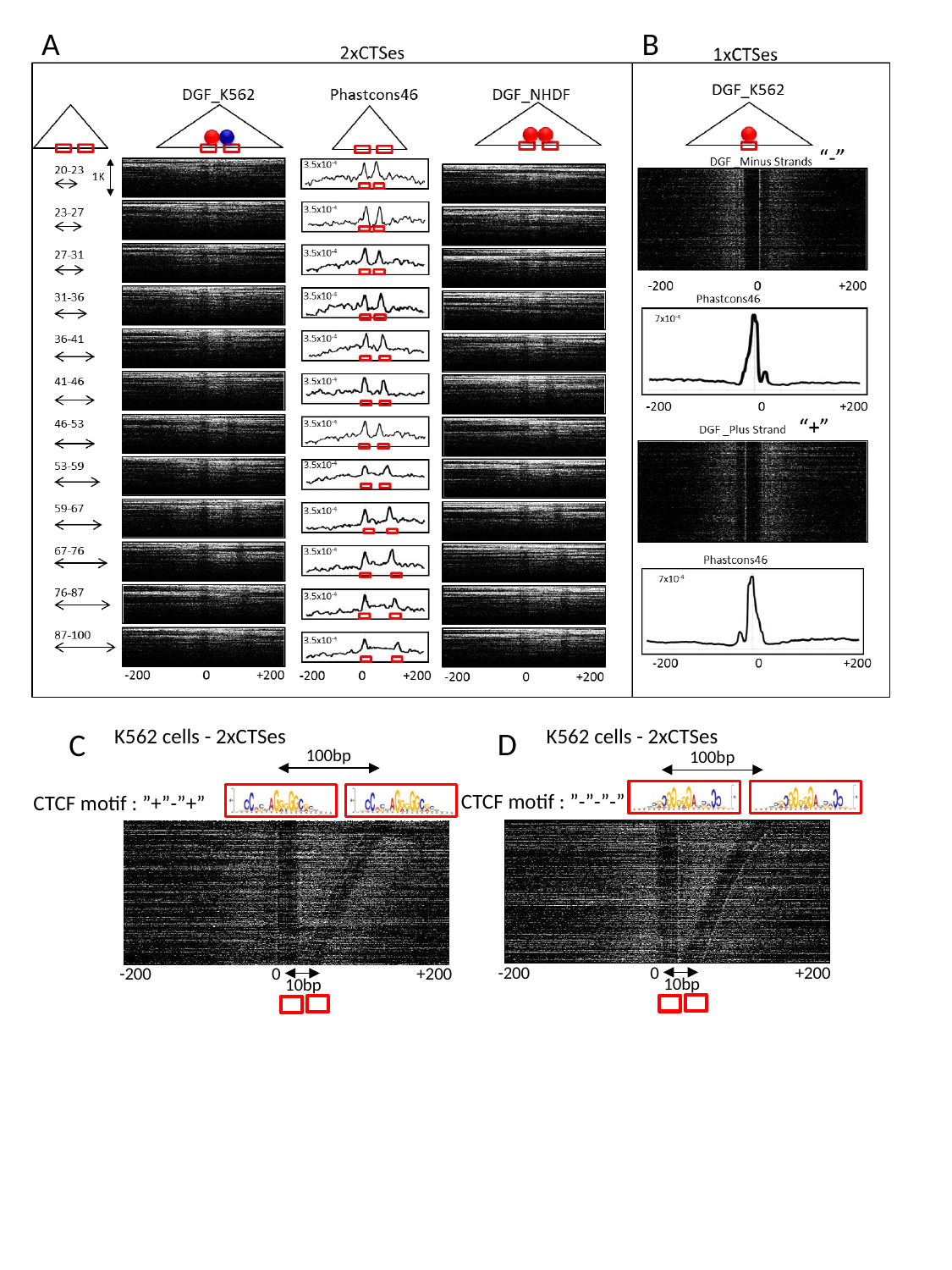

A
B
K562 cells - 2xCTSes
K562 cells - 2xCTSes
D
C
100bp
100bp
CTCF motif : ”-”-”-”
CTCF motif : ”+”-”+”
-200 0 +200
-200 0 +200
10bp
10bp

Supplement: Additional file 6: Fig. S6. — Comparison of DNaseI digital genomic footprinting (DGF) and phastConss46 conservation score at 2xCTSes and 1xCTSes. a Heatmaps show DNaseI cleavage density mapped in K562 and NHDF cells at 2xCTSes. 2xCTSes (CTCF&BORIS bound regions in K562 cells) were sorted into 12 different groups (1000 genomic regions in each group) depending on the distance between two CTCF motifs. The distance is shown by double arrows and the number of base pairs between two CTCF motifs. The same genomic coordinates were used to plot DNaseI cleavage density mapped in K562 and NHDF cells and for the analysis of average phastCons46 conservation score (shown in the middle). The schematic presentation of 2xCTSes with two CTCF motifs (red boxes) under one ChIP-seq peak and the proposed occupancy of 2xCTSes by CTCF (red circle) and BORIS (blue circle) in K562 and NHDF cells are shown at the top of panels. The two CTCF motifs were not sorted based on plus or minus strand. The tag density data were collected within a 400-bp window around the first (left) 20-bp CTCF core motifs (0) under a single CTCF ChIP-seq peak. b Heatmap shows DNaseI cleavage density mapped in K562 cells at 1xCTSes with CTCF motif on minus (upper panel) and plus (lower panel) strands. The average phastCons conservation score at the same regions as in DNAseI panels is shown below. c, d Heatmaps show DNaseI cleavage density mapped in K562 cells at 2xCTSes. 2xCTSes were separated into two groups with both CTCF motifs located on the plus strand (c) or minus strand (d). The distance between two CTCF motifs varies from 10 (bottom) to 100 bp (top). (PPTX 1337 kb) [file 13059_2015_736_MOESM6_ESM.pptx]
