# Supplementary material for: Comparative analyses of CTCF and BORIS occupancies uncover two distinct classes of CTCF binding genomic regions
Source: Genome Biol. 2015 Aug 14;16(1):161. doi: 10.1186/s13059-015-0736-8 (PMC4562119; doi:10.1186/s13059-015-0736-8)
Supplement: Additional file 7: Fig. S7. — Epigenetic profile of two classes of CTCF binding regions in BORIS-positive cells (K562). a Heatmaps demonstrate the association of CTCF&BORIS bound regions with active promoters (H3K4me3 and RNAPII, ENCODE) and enhancers (H3K27ac, ENCODE) mapped in K562 cells. The tag density was subjected to k-means ranked clustering with five clusters expected. b Genome browser view of two examples of Super Enhancers mapped in K562 cells. H3K27ac and p300 tracks were adopted from ENCODE. c Scatter plots show the overlapping of CTCF-only and CTCF&BORIS bound regions (left panel) and BORIS-only and CTCF&BORIS bound regions (right panel) with ChIP-seq data available for K562 cells by ENCODE. The dots represent the percentage of CTCF-only, BORIS-only (x-axis), and CTCF&BORIS (y-axis) bound regions overlapping with each factor mapped by ChIP-seq in K562 cells. The red dots are labeled with the name of the factor mapped by ChIP-seq. d The percentage of BORIS-only, CTCF&BORIS and CTCF-only bound regions (y-axis) overlapping with each factor (x-axis). e Heatmap demonstrates the enrichment of RNAPII, CAGEs, H3K4me3, H2AZ, H3K27ac, ZNF143, and SMC3 ChIP-seq data (ENCODE data) at CTCF&BORIS bound regions in contrast to CTCF-only bound regions. The tag density of ChIP-seq data was collected within a 10-kb window around the summit of CTCF peaks mapped in K562 cells. The collected data were subjected to k-means clustering using linear normalization. f Average tag density (tags/10 million) of multiple factors mapped by ChIP-seq in K562 cells (ENCODE data) across BORIS-only (blue), CTCF-only (red) and CTCF&BORIS (purple) bound regions mapped in K562 cells. The names of factors used in ChIP-seq are labeled on the top of the each plot. g Histogram shows the genomic distribution of CTCF and BORIS occupancy at promoters plus the 5’ UTR (3 kb up- and downstream of TSSs), gene bodies, and intergenic regions. (PPTX 680 kb) [file 13059_2015_736_MOESM7_ESM.pptx]

## Slide 1
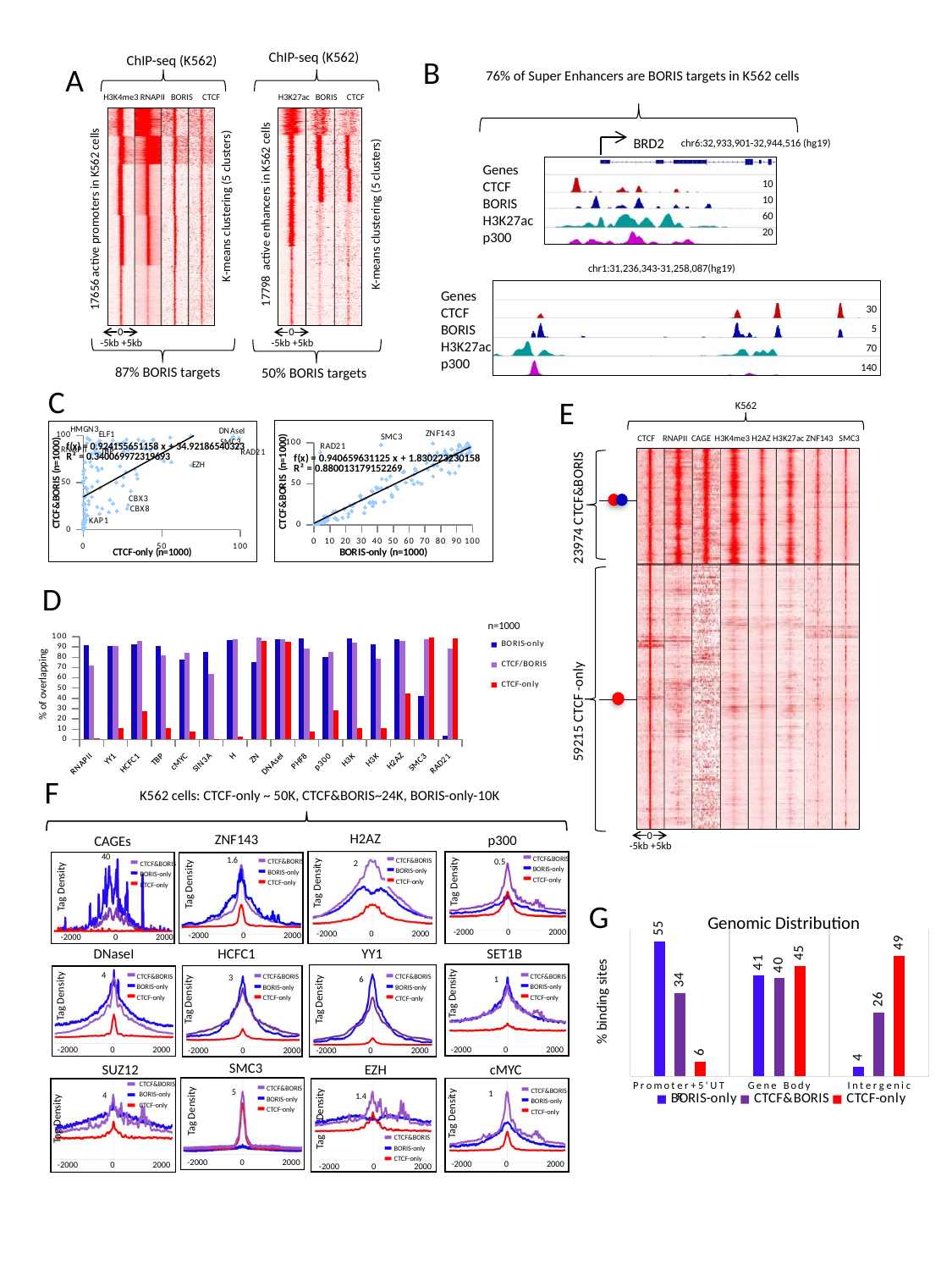

ChIP-seq (K562)
ChIP-seq (K562)
A
H3K4me3 RNAPII BORIS CTCF
H3K27ac BORIS CTCF
K-means clustering (5 clusters)
K-means clustering (5 clusters)
17798 active enhancers in K562 cells
17656 active promoters in K562 cells
87% BORIS targets
50% BORIS targets
B
76% of Super Enhancers are BORIS targets in K562 cells
BRD2
chr6:32,933,901-32,944,516 (hg19)
Genes
CTCF
BORIS
H3K27ac
p300
10
10
60
20
chr1:31,236,343-31,258,087(hg19)
Genes
CTCF
BORIS
H3K27ac
p300
30
5
70
140
0
-5kb +5kb
0
-5kb +5kb
C
E
K562
59215 CTCF -only 23974 CTCF&BORIS
### Chart
| Category | |
|---|---|
### Chart
| Category | |
|---|---|CTCF RNAPII CAGE H3K4me3 H2AZ H3K27ac ZNF143 SMC3
D
n=1000
### Chart
| Category | BORIS-only | CTCF/BORIS | CTCF-only |
|---|---|---|---|
| RNAPII | 91.4 | 71.8 | 1.0 |
| YY1 | 90.8 | 91.1 | 10.9 |
| HCFC1 | 92.3 | 95.9 | 27.6 |
| TBP | 90.7 | 82.3 | 10.8 |
| cMYC | 78.2 | 84.3 | 7.6 |
| SIN3A | 85.2 | 63.8 | 0.6 |
| HMGN3 | 96.3 | 97.4 | 2.7 |
| ZNF143 | 75.0 | 99.0 | 95.6 |
| DNAseI | 97.2 | 97.6 | 95.0 |
| PHF8 | 98.6 | 88.5 | 7.5 |
| p300 | 80.7 | 85.2 | 28.5 |
| H3K4me3 | 98.3 | 94.5 | 10.7 |
| H3K27ac | 92.5 | 78.3 | 10.7 |
| H2AZ | 97.7 | 95.8 | 44.6 |
| SMC3 | 42.3 | 97.4 | 99.2 |
| RAD21 | 4.0 | 88.4 | 98.2 |% of overlapping
F
K562 cells: CTCF-only ~ 50K, CTCF&BORIS~24K, BORIS-only-10K
0
-5kb +5kb
H2AZ
ZNF143
p300
CAGEs
CTCF&BORIS
BORIS-only
CTCF-only
0.5
Tag Density
 -2000 0 2000
40
CTCF&BORIS
BORIS-only
CTCF-only
Tag Density
 -2000 0 2000
CTCF&BORIS
BORIS-only
CTCF-only
2
Tag Density
 -2000 0 2000
1.6
CTCF&BORIS
BORIS-only
CTCF-only
Tag Density
 -2000 0 2000
G
### Chart
| Category | BORIS-only | CTCF&BORIS | CTCF-only |
|---|---|---|---|
| Promoter+5'UTR | 55.0 | 34.0 | 6.0 |
| Gene Body | 41.0 | 40.0 | 45.0 |
| Intergenic | 4.0 | 26.0 | 49.0 |Genomic Distribution
% binding sites
DNaseI
HCFC1
YY1
SET1B
CTCF&BORIS
BORIS-only
CTCF-only
1
Tag Density
 -2000 0 2000
4
CTCF&BORIS
BORIS-only
CTCF-only
Tag Density
 -2000 0 2000
CTCF&BORIS
BORIS-only
CTCF-only
3
Tag Density
 -2000 0 2000
CTCF&BORIS
BORIS-only
CTCF-only
6
Tag Density
 -2000 0 2000
SMC3
EZH
SUZ12
cMYC
CTCF&BORIS
BORIS-only
CTCF-only
4
Tag Density
 -2000 0 2000
CTCF&BORIS
BORIS-only
CTCF-only
5
Tag Density
 -2000 0 2000
1.4
Tag Density
CTCF&BORIS
BORIS-only
CTCF-only
 -2000 0 2000
CTCF&BORIS
BORIS-only
CTCF-only
1
Tag Density
 -2000 0 2000
