# Supplementary material for: Comparative analyses of CTCF and BORIS occupancies uncover two distinct classes of CTCF binding genomic regions
Source: Genome Biol. 2015 Aug 14;16(1):161. doi: 10.1186/s13059-015-0736-8 (PMC4562119; doi:10.1186/s13059-015-0736-8)
Supplement: Additional file 8: Fig. S8. — Epigenetic profile of two classes of CTCF binding regions in BORIS-negative cells (GM12878). a Average tag density (tags/10 million) of CTCF, RNAPII, DNaseI digestion, H3K4me3, H3K27ac, and RAD21 (mapped by ChIP-seq in GM12878 cells, ENCODE data) at 2xCTSes (blue) and 1xCTSes (red). The data were normalized to the number of mapped reads and binding regions. b Upper panel: scatter plot shows overlapping of 2xCTSes (y-axis) and 1xCTSes (x-axis) with multiple ENCODE data available for GM12878. Similar to CTCF&BORIS and CTCF-only bound regions in K562 (Fig. S7c, left panel in Additional file 7), no correlation (R2 = 0.41) was found between 2xCTSes and 1xCTSes with respect to co-occupancy with the majority of transcription factors, histone modifications, and chromatin remodeling factors. Middle panel: scatter plot shows an overlapping of 1xCTSes mapped in K562 (y-axis) and in GM12878 cells (x-axis) with ENCODE data for K562 and GM12878 cells, respectively. 1xCTSes of GM12878 and K562 cells were correlated with each other (R2 = 0.98) with respect to co-occupancy with the majority of ENCODE data common for the two cell lines. Lower panel: scatter plot shows the overlapping of 2xCTSes in K562 (y-axis) and in GM12878 (x-axis) with ENCODE data for K562 and GM12878 cells (R2 = 0.93), respectively. (PPTX 220 kb) [file 13059_2015_736_MOESM8_ESM.pptx]

## Slide 1
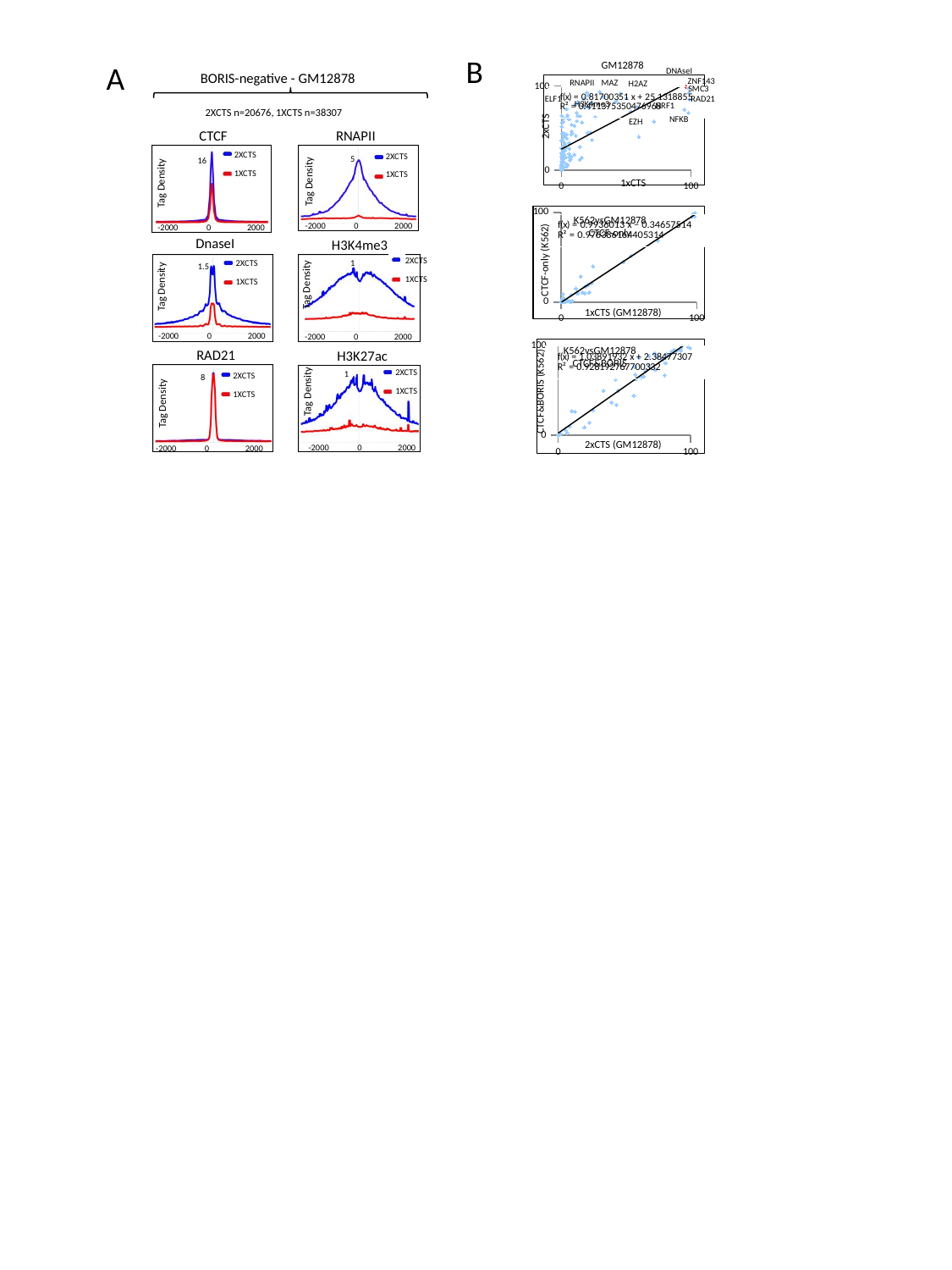

B
A
GM12878
DNAseI
BORIS-negative - GM12878
2XCTS n=20676, 1XCTS n=38307
CTCF
2XCTS
1XCTS
16
Tag Density
-2000 0 2000
RNAPII
5
Tag Density
-2000 0 2000
2XCTS
1XCTS
DnaseI
1.5
Tag Density
-2000 0 2000
H3K4me3
1
Tag Density
-2000 0 2000
2XCTS
1XCTS
2XCTS
1XCTS
RAD21
H3K27ac
1
Tag Density
-2000 0 2000
2XCTS
1XCTS
2XCTS
1XCTS
8
Tag Density
-2000 0 2000
ZNF143
RNAPII
MAZ
H2AZ
### Chart
| Category | |
|---|---|SMC3
RAD21
ELF1
H3K4me3
NRF1
NFKB
EZH
2xCTS
1xCTS
### Chart
| Category | |
|---|---|K562vsGM12878
CTCF-only
CTCF-only (K562)
1xCTS (GM12878)
K562vsGM12878
CTCF&BORIS
### Chart
| Category | |
|---|---|CTCF&BORIS (K562)
2xCTS (GM12878)
