# Supplementary material for: Comparative analyses of CTCF and BORIS occupancies uncover two distinct classes of CTCF binding genomic regions
Source: Genome Biol. 2015 Aug 14;16(1):161. doi: 10.1186/s13059-015-0736-8 (PMC4562119; doi:10.1186/s13059-015-0736-8)
Supplement: Additional file 9: Fig. S9. — BORIS occupancy in cancer cells recapitulates the BORIS occupancy in germ cells. a Average tag density of CTCF (red) and BORIS (blue) occupancies mapped in OVCAR8 and Delta47 cells across the conserved mouse binding regions (upper panel). b Gene tracks representing the examples of conserved BORIS-only, CTCF-only and CTCF&BORIS bound regions in germline (round spermatids) and cancer cells (K562). (PPTX 104 kb) [file 13059_2015_736_MOESM9_ESM.pptx]

## Slide 1
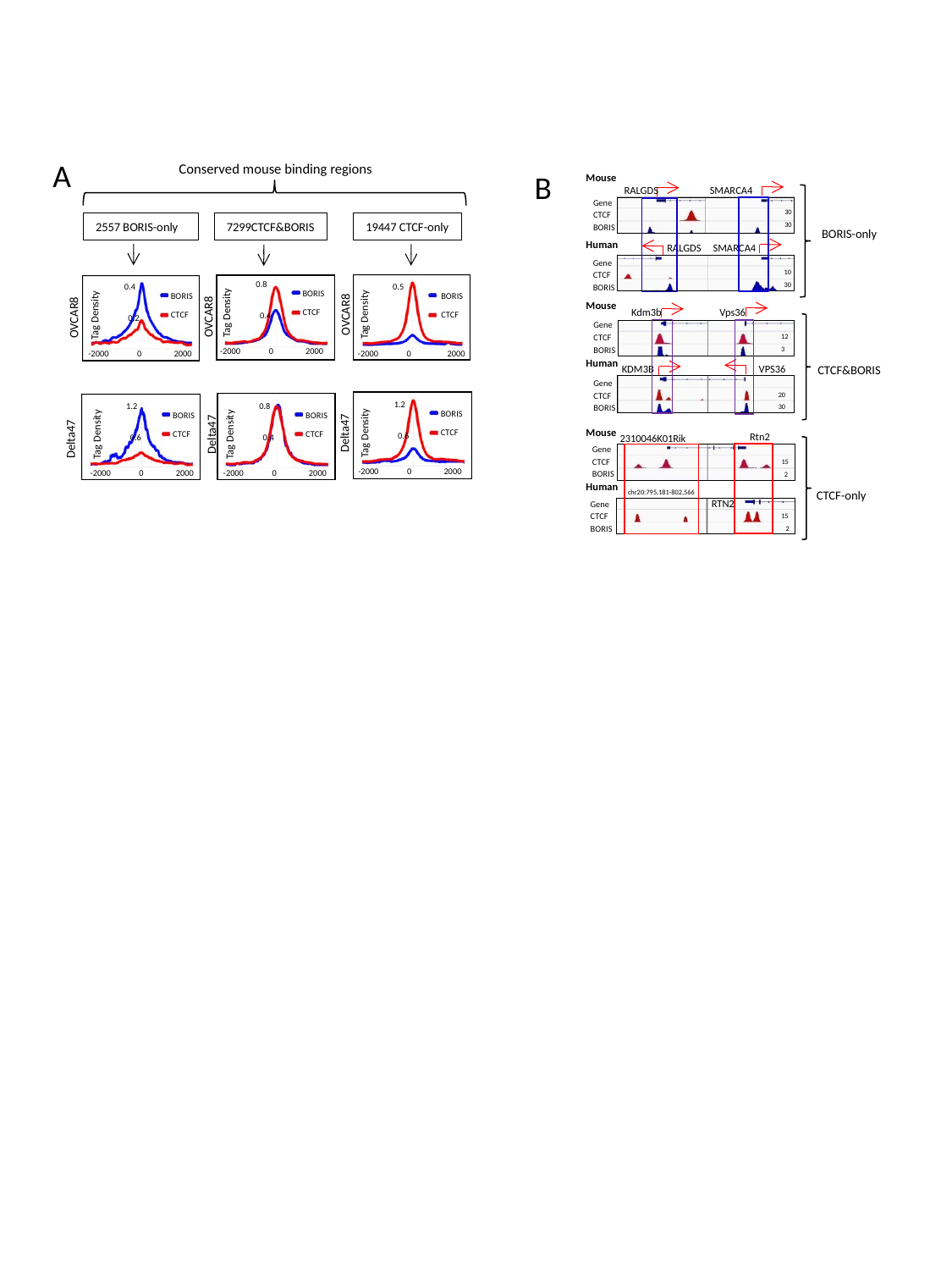

A
Conserved mouse binding regions
B
Mouse
RALGDS SMARCA4
Gene
CTCF
BORIS
30
30
BORIS-only
Human
RALGDS SMARCA4
Gene
CTCF
BORIS
10
30
Mouse
Kdm3b Vps36
Gene
CTCF
BORIS
12
3
Human
CTCF&BORIS
KDM3B VPS36
Gene
CTCF
BORIS
20
30
Mouse
Rtn2
2310046K01Rik
Gene
CTCF
BORIS
15
 2
Human
CTCF-only
chr20:795,181-802,566
Gene
CTCF
BORIS
RTN2
15
 2
2557 BORIS-only
7299CTCF&BORIS
19447 CTCF-only
0.8
 0.4
BORIS
CTCF
Tag Density
-2000 0 2000
0.4
 0.2
BORIS
CTCF
Tag Density
-2000 0 2000
0.5
BORIS
CTCF
Tag Density
-2000 0 2000
OVCAR8
OVCAR8
OVCAR8
1.2
 0.6
BORIS
CTCF
Tag Density
-2000 0 2000
1.2
 0.6
BORIS
CTCF
Tag Density
-2000 0 2000
0.8
 0.4
BORIS
CTCF
Tag Density
-2000 0 2000
Delta47
Delta47
Delta47
