# Supplementary material for: Comparative analyses of CTCF and BORIS occupancies uncover two distinct classes of CTCF binding genomic regions
Source: Genome Biol. 2015 Aug 14;16(1):161. doi: 10.1186/s13059-015-0736-8 (PMC4562119; doi:10.1186/s13059-015-0736-8)
Supplement: Additional file 10: Fig. S10. — BORIS is involved in transcriptional program of cancers. a The sequence recognized by ZFN and cleaved by Fok I is shown by black and red letters, respectively. b Surveyor assay (CEL-I) for ZFN-induced mutations in the BORIS gene. The proportions of wild-type and mutant alleles are shown as a table at the bottom of the gel. c Surveyor assay shows ten single-cell clones with the mutated BORIS locus. d Western blot shows the level of BORIS protein in wild type (wt) and ten mutant clones from panel (c). e K562 cells transfected with ZFN produced at least ten times less colonies in soft agar compared with control. f Western blot shows the level of BORIS protein in K562 before (0) and after phorbol 12-myristate 13-acetate (PMA) treatment. Wright–Giemsa staining analysis of K562 transfected with either control vector or ZFN in comparison with K562 treated with PMA for 3 days. Both transfection of K562 with ZFN and treatment with PMA showed morphologic changes related to megakaryocytic differentiation. h Upper panel: ChIP-seq and RNA-seq tracks show CTCF and BORIS occupancy at the WISP2 gene and dowregulation of WISP2 expression upon BORIS induction in MCF7 cells. Lower panel: RNA-seq RPKM values for the WISP2 gene expression. i Downregulation of inflammatory response pathway in response to stable BORIS expression in MCF7 cells. k ChIP-seq tracks show CTCF and BORIS occupancy at GAL3ST1, FOXA3, and PRAME loci in NHDF, OVCAR8, Delta47, and K562 cells. The tracks are labeled with the molecules against which antibodies were directed and cell lines used in ChIP-seq. RNA-Seq tracks demonstrate gene expression in BORIS-positive cells (K562) in contrast to BORIS-negative cells (NHEK and NHDF). l Quantitative PCR analysis of GAL3ST1, FOXA3, and PRAME gene expression in BORIS-positive cells (Testes, K562, OVCAR8, Delta47) and BORIS-negative cells (GM12878, NHDF, NHEK). (PPTX 1328 kb) [file 13059_2015_736_MOESM10_ESM.pptx]

## Slide 1
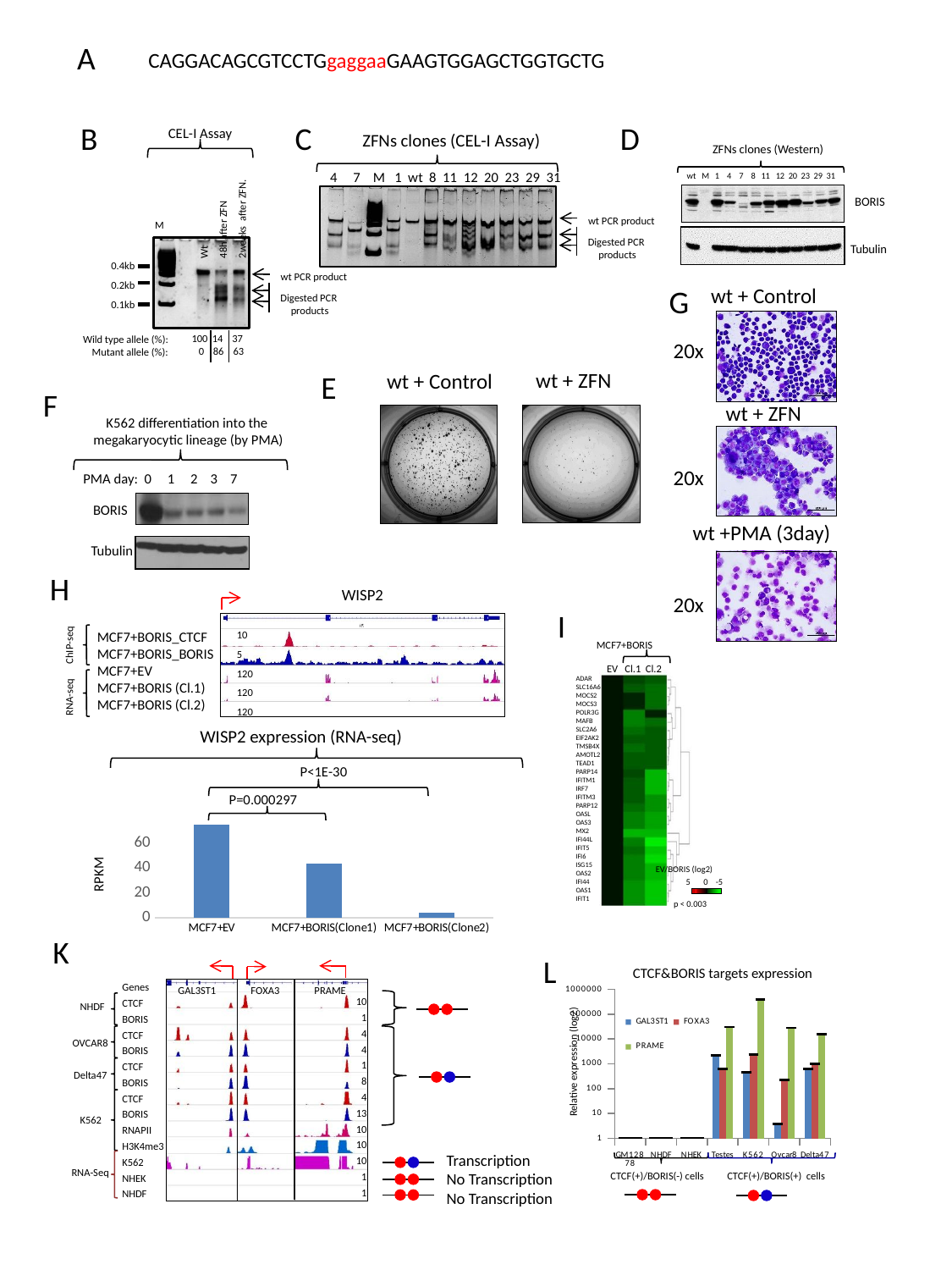

A
CAGGACAGCGTCCTGgaggaaGAAGTGGAGCTGGTGCTG
B
C
D
CEL-I Assay
ZFNs clones (CEL-I Assay)
wt PCR product
Digested PCR
 products
 4 7 M 1 wt 8 11 12 20 23 29 31
ZFNs clones (Western)
wt M 1 4 7 8 11 12 20 23 29 31
BORIS
Tubulin
Wt
48h after ZFN
2weeks after ZFN.
M
0.4kb
0.2kb
0.1kb
wt PCR product
G
wt + Control
Digested PCR
 products
 100 14 37
 0 86 63
Wild type allele (%):
Mutant allele (%):
20x
20x
20x
wt + ZFN
E
wt + Control
F
wt + ZFN
K562 differentiation into the
megakaryocytic lineage (by PMA)
 PMA day: 0 1 2 3 7
BORIS
Tubulin
wt +PMA (3day)
H
WISP2
10
5
120
120
120
MCF7+BORIS_CTCF
MCF7+BORIS_BORIS
MCF7+EV
MCF7+BORIS (Cl.1)
MCF7+BORIS (Cl.2)
ChIP-seq
RNA-seq
I
MCF7+BORIS
EV Cl.1 Cl.2
ADAR
SLC16A6
MOCS2
MOCS3
POLR3G
MAFB
SLC2A6
EIF2AK2
TMSB4X
AMOTL2
TEAD1
PARP14
IFITM1
IRF7
IFITM3
PARP12
OASL
OAS3
MX2
IFI44L
IFIT5
IFI6
ISG15
OAS2
IFI44
OAS1
IFIT1
EV/BORIS (log2)
5 0 -5
p < 0.003
WISP2 expression (RNA-seq)
P<1E-30
P=0.000297
### Chart
| Category | |
|---|---|
| MCF7+EV | 76.2022 |
| MCF7+BORIS(Clone1) | 43.5943 |
| MCF7+BORIS(Clone2) | 4.2456 |RPKM
K
Genes
CTCF
BORIS
CTCF
BORIS
CTCF
BORIS
CTCF
BORIS
RNAPII
H3K4me3
K562
NHEK
NHDF
GAL3ST1 FOXA3 PRAME
10
1
4
4
1
8
4
13
10
10
10
1
1
NHDF
OVCAR8
Delta47
K562
Transcription
No Transcription
No Transcription
RNA-Seq
L
CTCF&BORIS targets expression
### Chart
| Category | GAL3ST1 | FOXA3 | PRAME |
|---|---|---|---|
| GM12878 | 1.0 | 1.0 | 1.0 |
| NHDF | 1.0 | 1.0 | 1.0 |
| NHEK | 1.0 | 1.0 | 1.0 |
| Testes | 2219.17696134449 | 645.0496201907289 | 30565.622593846896 |
| K562 | 454.14724979326246 | 2341.535182613111 | 393733.97099440434 |
| Ovcar8 | 3.8212544662772934 | 224.9897291629876 | 28490.092132458325 |
| Delta47 | 617.6237126406452 | 1028.5472342617397 | 15813.692615505455 |Relative expression (log2)
CTCF(+)/BORIS(-) cells CTCF(+)/BORIS(+) cells
