# Supplementary material for: Comparative analyses of CTCF and BORIS occupancies uncover two distinct classes of CTCF binding genomic regions
Source: Genome Biol. 2015 Aug 14;16(1):161. doi: 10.1186/s13059-015-0736-8 (PMC4562119; doi:10.1186/s13059-015-0736-8)
Supplement: Additional file 12: Fig. S11. — CTCF and BORIS bound regions enriched at the anchors of cell-type specific transcriptional loops in K562 cells compared with MCF7 cells. a Heatmap shows the enrichment of BORIS, CTCF, and RNAPII occupancy in K562 cells at the left and right anchors of K562-specific loops. b Heatmap shows the enrichment of CTCF and RNAPII occupancy in MCF7 cells at the anchors of K562-specific loops, but the interactions mediated by CTCF were different in MCF7 cells compared with K562 cells. c Genomic view of K562-specific long-range chromatin interactions at the COMMD7-DNMT3B-MAPRE1 locus. The tracks are labeled with the molecules against which antibodies were directed in ChIP-seq in both K562 and MCF7 cells. ChIA-PET data are presented as red lines with bold anchors and summarized by brackets on the top of the gene tracks. d Model of cluster and single CTCF binding classes and their modulation by BORIS in BORIS-positive cells. The model is based on ChIP-seq and ChIA-PET data for K562 and MCF7 cells at the DNMT3B locus (c), but also observed in TP53, FOXA3, PRAME, KDM3B, MDM2, BBC3 and other loci. (PPTX 209 kb) [file 13059_2015_736_MOESM12_ESM.pptx]

## Slide 1
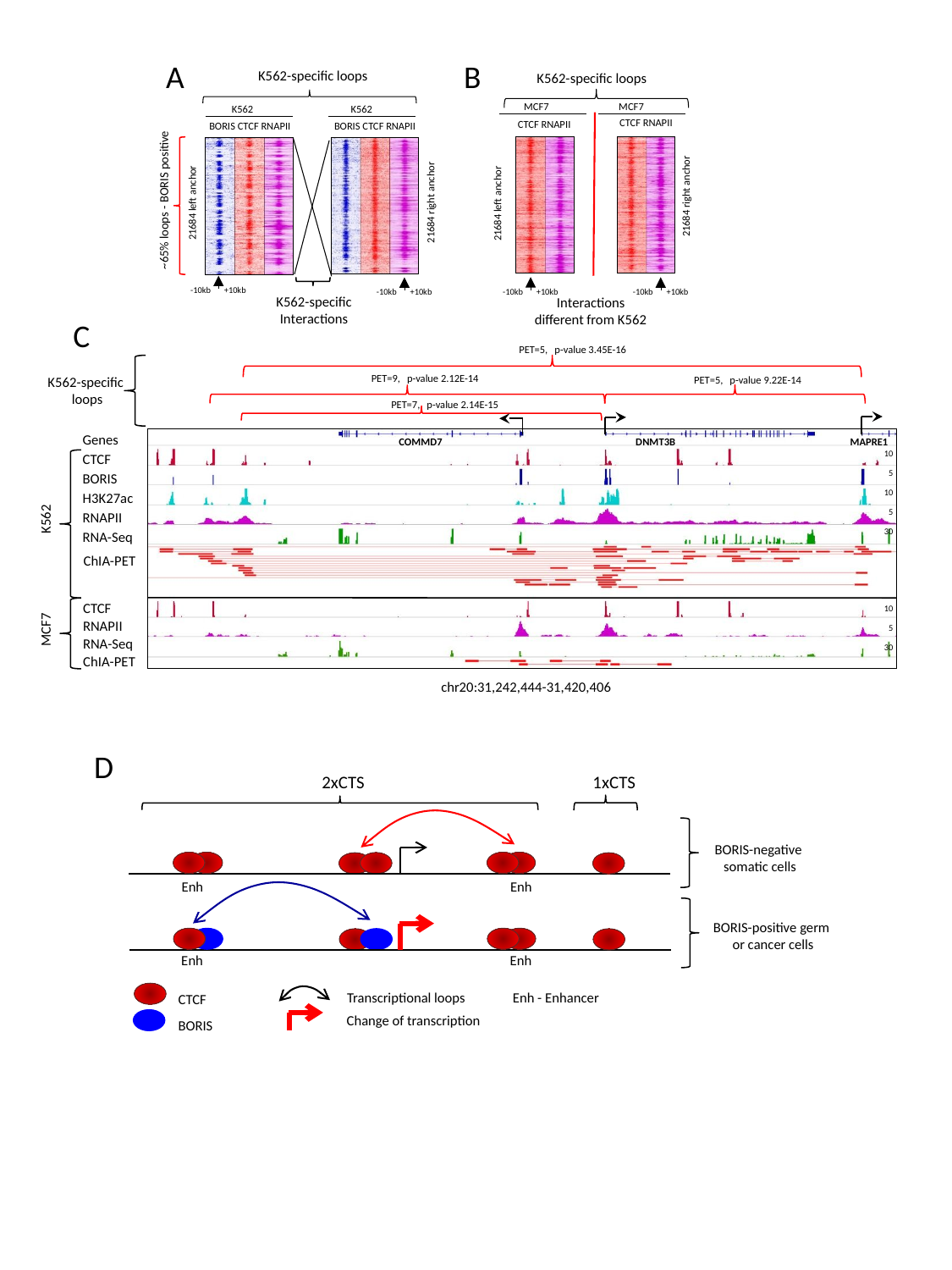

A B
K562-specific loops
K562 K562
BORIS CTCF RNAPII
BORIS CTCF RNAPII
~65% loops - BORIS positive
21684 right anchor
21684 left anchor
-10kb +10kb
-10kb +10kb
K562-specific
Interactions
K562-specific loops
MCF7 MCF7
CTCF RNAPII
 CTCF RNAPII
21684 right anchor
21684 left anchor
-10kb +10kb
-10kb +10kb
Interactions
different from K562
C
PET=5, p-value 3.45E-16
PET=9, p-value 2.12E-14
K562-specific
loops
PET=5, p-value 9.22E-14
PET=7, p-value 2.14E-15
Genes
CTCF
BORIS
H3K27ac
RNAPII
RNA-Seq
COMMD7 DNMT3B MAPRE1
10
5
10
5
30
10
5
30
ChIA-PET
MCF7 K562
CTCF
RNAPII
RNA-Seq
ChIA-PET
chr20:31,242,444-31,420,406
D
2xCTS 1xCTS
BORIS-negative
somatic cells
Enh Enh
BORIS-positive germ
or cancer cells
Enh Enh
Transcriptional loops Enh - Enhancer
CTCF
BORIS
Change of transcription
