# Supplementary material for: Comparative analyses of CTCF and BORIS occupancies uncover two distinct classes of CTCF binding genomic regions
Source: Genome Biol. 2015 Aug 14;16(1):161. doi: 10.1186/s13059-015-0736-8 (PMC4562119; doi:10.1186/s13059-015-0736-8)
Supplement: Additional file 13: Table S9 — The primers used in the study for amplification of EMSA probes and for quantitative PCR. (DOCX 17 kb) [file 13059_2015_736_MOESM13_ESM.docx]

| **Primers for amplification of probes used for EMSA and for qPCR** | |
| --- | --- |
| **Figure1B, S1D** | |
| WT1 (Fragment 5) | 5’ gtctcctcgccgcgatcctggacttcctc 3’ |
|  | 5’ cacgtcggagcccatttgctgcggctcag 3’ |
| H19/IGF2 ICR | 5’ CGGCGCCTGGCTTGCGGGACCCGG 3’ |
|  | 5’ GAATATTTCTGGAGGCTTCCCCTTC 3’ |
| **Figure 2C; 3E,F; S5B,C** | |
| FOXA3 promoter (CTCF&BORIS) | 5’ GTCGCTGCGGGCAGCTCCCTCACTCCTGAATCAC 3’  5’ GAGGCGGCCACGCTTTATAGCCGGGACACC 3’ |
| KDM3B promoter (CTCF&BORIS) | 5’ GGGAAGCTGAAGGACCCGCCCACTTCC 3’ |
|  | 5’ CACCAACCACCGGCGCAGCGCAGCGCC 3’ |
| TP53 promoter (CTCF&BORIS) | 5’ GAGCTCGATAATAAATATTTTTTGAATGAG 3’ |
|  | 5’ CTGAACGCTTCTATCTTGGCGAGAAGC 3’ |
| CTCF&BORIS (chr17) | 5’ GACTTTTGGTAGCACTCAGTTCCTGGATTCAG 3’ |
|  | 5’ CCCCTTTTACTGCTAGTTATGAAAGTTTCTC 3’ |
| IRF2BP1 (CTCF&BORIS) | 5’ CTCTGAGCCTCAGTACTTTTATCTGGAAAACG 3’ |
|  | 5’ CCTTAGTTGTGTGAGATGCTGCGCCCCCTTTG 3’ |
| CTCF-only (chr2, Intergenic) | 5’ CTATGATCATTCCCTGTGATTGAAAC 3’ |
|  | 5’ CAACATTCTACAGAATACTTCGGGAAAATAG 3’ |
| CTCF-only (EFCAB5) | 5’ GTTGCCCAGGCTGGTCTCAAACTC 3’ |
|  | 5’ CAAGGAGGTACAAGGAAATCATTCTGAC 3’ |
| CTCF-only (chr4, Intergenic) | 5’ GCACAATATACAGAATAATTTTACAAC 3’ |
|  | 5’ CGAACTGTTGCCTCTTTCTTGTTCCAGCCTG 3’ |
| CTCF-only (H19) | 5’ CGGCGCCTGGCTTGCGGGACCCGG 3’ |
|  | 5’ GAATATTTCTGGAGGCTTCCCCTTC 3’ |
| **Figure S3, Panel A** | |
| TP53 promoter (CTCF&BORIS) | 5’ GAGCTCGATAATAAATATTTTTTGAATGAG 3’ |
|  | 5’ CTGAACGCTTCTATCTTGGCGAGAAGC 3’ |
| H19/IGF2 ICR (CTCF-only) | 5’ CGGCGCCTGGCTTGCGGGACCCGG 3’ |
|  | 5’ GAATATTTCTGGAGGCTTCCCCTTC 3’ |
| BMI promoter (BORIS-only) | 5’ GCGCCCCGGGAGGAGGCGGGCGGCCG 3’ |
|  | 5’ CCCCTCCCCTCCGCGCCGCGCGCCCGGC 3’ |
| PROB1 (CTCF&BORIS, BORIS-only) | 5’ GGTGGTGTCTTCGGTTTGGGTGCTATC 3’  5’ GCGAGTGCGAGCGCGCGGCCCTG 3’ |
| **Figure S3, Panel B** | |
| CTCF-only (chr3) | 5’ CACCCATATCTCACTTTCTTTGCAAG 3’ |
|  | 5’ CAGCAGCATGAAGAGGGCTGTGGATCAGG 3’ |
| CTCF-only (chr19) | 5’ GAATTAAGGTATCACAGAGGTAGGGTC 3’ |
|  | 5’ CAGGAGTTTTTCACCCCACCCAGAGTTTTAAG 3’ |
| CTCF-only (chr11) | 5’ CACATTTTAGTATTATAAGCAGTAACAGTTG 3’ |
|  | 5’ CATGACACGGCAGTGATGCCTAATTTTTTAGAAAGTTG 3’ |
| CTCF-only (chr5) | 5’ CTCTGCTCTTCTCTGGAAGCAGGTATCTG 3’ |
|  | 5’ CTCCTCATAGGTGCTCTTCATATAGTTTATC 3’ |
| CTCF-only (chr20) | 5’ GTGAAGATAAAGGTGGTGATGAGTTCCTC 3’ |
|  | 5’ CACCAGAGATCTCTTAATACGACCACCAAACAC 3’ |
| **Figure S3, Panel C** | |
| BORIS-only (ZNF258) | 5’ GCTGCGTGCGGGCGGGGCACGGTGCAG 3’ |
|  | 5’ CCCCCCGGGCCCGCAGGAAGCCCCCCGCCC 3’ |
| BORIS-only (BOP1) | 5’ CGGCCACGTGCCCGCCGGGCCCTCTAGGGAC 3’ |
|  | 5’ CCTTCCCTCCACCCGCCAATCGGGGCC 3’ |
| BORIS-only (RNF220) | 5’ GCGCGACGGCGGCAGATCACGTGATTAGG 3’ |
|  | 5’ CCTACTTCCTCCCTCCCGCCGGCCCCTCTG 3’ |
| BORIS-only (CLN5) | 5’ GTGTCATGCGCCGGAACCTGCGCTTGGGGC 3’ |
|  | 5’ CCGGGACCAGCCCGGAACCACCGCGAGC 3’ |
| BORIS-only (chr11) | 5’ CGGCCTTGGCCCCCACCGCCCTCATCCCG 3’ |
|  | 5’ CGCCCGTCGCGGCCGGGCTGCAGGCGCGG 3’ |
| **Figure S5, panel A** | |
| DNMT3B promoter (CTCF&BORIS) | 5’ CACCCACTCCCGCTGCCCCGTCCGGCC 3’ |
|  | 5’ CCGGCGCCGATCGCCGAGCTAGGTTTACTTG 3’ |
| GAL3ST1 testis-specific promoter (CTCF&BORIS) | 5’ CACCCCGGCCCCAGCAGCCCCACTCACCAAG 3’ |
|  | 5’ GAGTGAATGAGTGAATGAGTGTACCAGTAATGG 3’ |
| MDM2 promoter  (CTCF&BORIS) | 5’ GAGCCCCGGACCCAATTGGCGGAAGC 3’ |
|  | 5’ GGGTCGCGGCCGCCCCTCGGGCTC 3’ |
| BIVM intron (CTCF&BORIS) | 5’ GCCCGTGGGCCCCCTTTAACAC 3’ |
|  | 5’ CCCCGAGTCCCCAGCCCGCAGAGCC 3’ |
| EFNA3 intron (CTCF&BORIS) | 5’ GGGAGAAGCCGGTCCCCACTCTCCC 3’ |
|  | 5’ GGAGCCAGGGACGTGACCACCCACAG 3’ |
| Chr13 (CTCF&BORIS) | 5’ GCAAGCCTGAGTCAGCTCGCGGCG 3’ |
|  | 5’ GCGTCCACTGTTTTCTTTAGCTGAAAACTAC 3’ |
| NUTD15 intron (CTCF&BORIS) | 5’ CGTCCGCGCTGACGCTGCGTCCTTTCTTG 3’ |
|  | 5’ GTAAGCTGTGAGAAACACACCTCACAGAC 3’ |
| IQGAP2 intron (CTCF-only) | 5’ GAGAAAATCAAATGGCCAGAATTG 3’ |
|  | 5’ CTGTACATGACCCAAGAGAATTAG 3’ |
| OBFC1 intron (CTCF-only) | 5’ CTCAGCTGTGGTGAAAACCACTTCCAAG 3’ |
|  | 5’ GTAGGTGTTCAGGAAGTCTATGG 3’ |
| INS_IGF2 intron (CTCF-only) | 5’ GCATGAGAGTCTTGGGAACCC 3’ |
|  | 5’ GACACTGTAGGGAGGCGTCCTGACTTTG 3’ |
| MPEG1 exon (CTCF-only) | 5’ GGGAGAGGCAGGTGAATGTCCTAAG 3’ |
|  | 5’ GGTGCACTAATCTACAAACAGCAGAAG 3’ |
| Chr12 Intergenic (CTCF-only) | 5’ GGGCTGCCCTGGGAGAGTGGACTC 3’ |
|  | 5’ CCCTCGCAAATATTTTGGAATGAATAAATTC 3’ |
| Chr18 Intergenic (CTCF-only) | 5’ CTTGGTGTGAGAATTCCAAGTGTG 3’ |
|  | 5’ GTACCTTGGCTGCCAAACATCTCCAG 3’ |
| Chr4 Intergenic (CTCF-only) | 5’ CTTTAGGCAAATCATTTAACCTCTG 3’ |
|  | 5’ CTAACGTGAAGTCATTTTCTAACTTGTG 3’ |
| **Real-Time PCR expression (Figure S1A, 6D,I, S10K)** | |
| VEGF | 5’ TGCCACCTCCATGTTTGATG 3’ |
|  | 5’ CCCCGACTCCTTACTTTTACTG 3’ |
| CD61 | 5’CAAGTGTGAATGTGGCAGC 3’ |
|  | 5’ TTTTCGTCATGTAGGGCTCC 3’ |
| CD44 | 5’ TGAGCATCGGATTTGAGACC 3’ |
|  | 5’ GTCATACTGGGAGGTGTTGG 3’ |
| MPL | 5’ TGCTTTACCTTGGACCTGAAG 3’  5’ CTCTTCCTCTTCGCAGTTCTC 3’ |
| THBS1 | 5’ CTCCCCTATGCTATCACAACG 3’ |
|  | 5’ AGGAACTGTGGCATTGGAG 3’ |
| GAL3ST1 | 5’ CTGATTCCAGAAGGTACCT 3’ |
|  | 5’ GAGGCGGGAGGATTGCTT 3’ |
| BBC3(PUMA) | 5’ CGACCTCAACGCACAGTAC 3’ |
|  | 5’ CCTAATTGGGCTCCATCTCG 3’ |
| FGFR4 | 5’ CTGGCTTAAGGATGGACAGG 3’ |
|  | 5’ CCACAGCGTTCTCTACCAG 3’ |
| EGR1 | 5’ CAGCACCTTCAACCCTCAG 3’ |
|  | 5’ AGTCGAGTGGTTTGGCTG 3’ |
| FOXA3 | 5’ GAAGATGGAGGCCCATGAC 3’ |
|  | 5’ GGATAGGGAGAGCTTAGAGGAT 3’ |
| PRAME | 5’ GGCTCTCTATGTGGACTCTTTATT 3’ |
|  | 5’ GAAAGCCGGCAGTTAGTTATTG 3’ |
| Mouse BORIS | 5´ CAAGTGCTCCCTGTGCAAGTAC 3´ |
|  | 5´ CGTGTGTGAGCGGATGTGA 3´ |
| Mouse CTCF | 5´ TCTCCAGATGAAGACTGAAGTCATG 3´ |
|  | 5´ TCCACTGCAGCCTCTGCTT 3´ |
| Human BORIS | 5’ gctgcggaaccatgttaacac 3’ |
|  | 5’ gccatgttgcagtcgttacact 3’ |
| Human CTCF | Hs00198081_m1 (Applied Biosystem, Life Technologies) |
| Human GAPDH | 5’ agccacatcgctcagagac 3’ |
|  | 5’ gcccaatacgaccaaatcc 3’ |
| Mouse GAPDH | 5´ AATGTGTCCGTCGTGGATCTGA 3´ |
|  | 5´ GATGCCTGCTTCACCACCTTCT 3´ |
